# Monitoring the Shear Behavior of Reinforced Concrete Beams Using Fiber Optic Sensors Installed in the Compression Zone

**DOI:** 10.3390/s26165226

**Published:** 2026-08-18

**Authors:** Johannes Rathgen, Vincent Oettel

**Affiliations:** Institute of Building Materials, Concrete Construction and Fire Safety (iBMB), Division of Concrete Construction, Technische Universität Braunschweig, Beethovenstraße 52, 38106 Braunschweig, Germany

**Keywords:** structural health monitoring, fiber optic sensors, strain gauges, experiments, shear behavior, concrete bridges

## Abstract

A variety of measurement systems are available for monitoring existing concrete bridges with deficiencies in shear capacity. In addition to established systems, fiber optic sensors (FOS) offer significant potential for structural health monitoring. However, FOSs are often installed in the tension zone, where crack formation may occur even under service loads, increasing the risk of sensor failure and potentially resulting in a loss of measurement capability. A promising approach to significantly reduce this risk is the installation of FOSs in the compression zone. However, it remains unclear whether measurements obtained from FOSs in the compression zone can be used to assess the load-bearing and deformation behavior of reinforced concrete beams and how they relate to measurements obtained in the tension zone. To address this question, shear tests were carried out on reinforced concrete beams with shear reinforcement ratios commonly used in practice. The experimental results demonstrate close agreement between measurements obtained from FOSs installed in the compression and tension zones under service loads. Furthermore, the findings and the characteristic strain profiles associated with shear and flexural failure provide a basis for assessing existing structures monitored using FOSs.

## 1. Introduction

**Figure 1:**
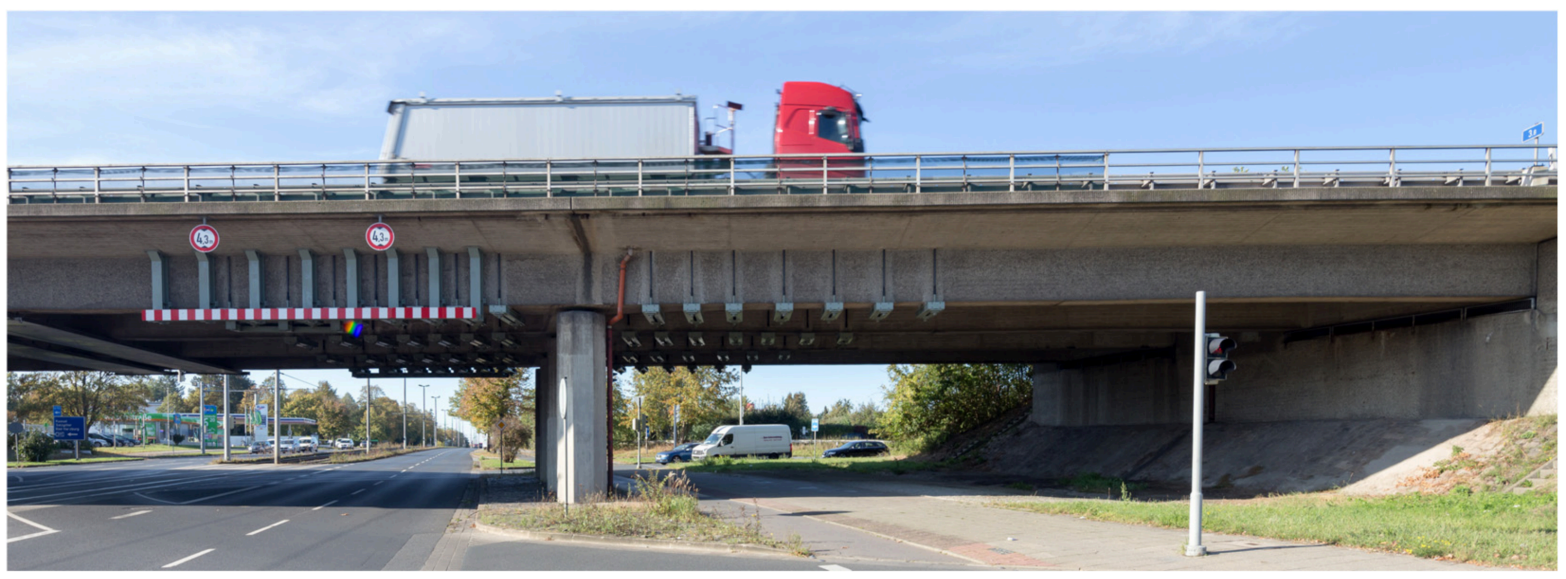
Prestressed concrete bridge in Germany retrofitted with steel clamps due to a calculated shear-capacity deficit.

A significant number of bridge structures in Germany, the USA, Canada, and worldwide exhibit deficiencies in load-bearing capacity due to their age, increased traffic loads and modifications in design codes [1,2,3,4]. In some cases, these deficiencies have led to the demolition and replacement of bridges. Especially when deficits in shear capacity are identified, replacement is often favored, as many older bridges do not have sufficient shear reinforcement to ensure a ductile failure mode. Moreover, due to the absence of reliable data on inherent material properties, it is often unfeasible to precisely forecast the occurrence and location of critical shear cracks and whether they pose a threat to the integrity of the entire structure. However, replacing all these bridges should be regarded as a last resort due to the high costs, lengthy approval processes, significant traffic disruptions, substantial material consumption, elevated CO_2_ emissions and limited availability of staff and machinery. In consideration of the aforementioned factors, it is evident that comprehensive replacement of the affected bridge structures is rendered almost infeasible in the near future. In addition to sustainable rapid construction methods for replacement bridges (e.g., [5,6]) and strengthening methods such as steel structures (Figure 1), carbon fiber-reinforced polymer strips (e.g., [7,8,9]), ultra-high-performance concrete overlay (e.g., [10,11,12,13]) or carbon concrete (e.g., [14]), monitoring and evaluation using fiber optic sensors (FOSs) in combination with a suitable monitoring concept ([15,16]) constitutes a promising solution that can be implemented in the short term to keep some of the bridges in service for longer and thereby gain time.

It has been shown that FOSs generally enable the detection of minimal strain changes with very high spatial resolution along the entire sensor length [17]. Consequently, they offer a promising tool for monitoring the shear behavior of existing reinforced and prestressed concrete bridges. Installing FOSs in the tension zone is common practice for monitoring load-bearing and deformation behavior (e.g., [18,19]). Additionally, FOSs can be installed in shear-critical regions near supports. However, these regions carry a high risk of sensor damage due to crack formation—with corresponding crack widths. This led to the novel idea of applying the FOS directly to the concrete surface in the compression zone of a bridge and using the measured strains to indirectly detect changes in load-bearing behavior and crack initiation. To date, the authors are aware of only one investigation—with a different focus—that has also applied FOSs in the compression zone [20].

To investigate this, experiments were carried out at the iBMB, Division of Concrete Construction at the TU Braunschweig, on reinforced concrete beams under monotonically increasing shear loading. Sensor fibers were placed in two distinct regions: on the concrete surface in the compression zone and on the lower longitudinal reinforcement in the tension zone. To validate the measurement data, strain gauges were also installed on the concrete surface and on the lower longitudinal reinforcement in both zones. This article presents the experimental program, outlines the sensor arrangements, and analyzes the resulting measurement data. First, a concise overview of established monitoring systems and fiber optic sensors is provided.

## 2. Measurement Systems for Monitoring Concrete Structures

### 2.1. Overview of Measurement Technologies

In addition to established systems, such as strain gauges, displacement transducers, and hydrostatic leveling systems [21], fiber optic sensors are increasingly employed to monitor the shear behavior of concrete structures [22]. Furthermore, digital image correlation (DIC) and other imaging techniques can be utilized. However, these are generally not designed for long-term use on bridges. The primary distinctions between the measurement systems pertain to the accuracy and the size of the areas that can be monitored.

### 2.2. Established Measurement Systems

**Figure 2:**
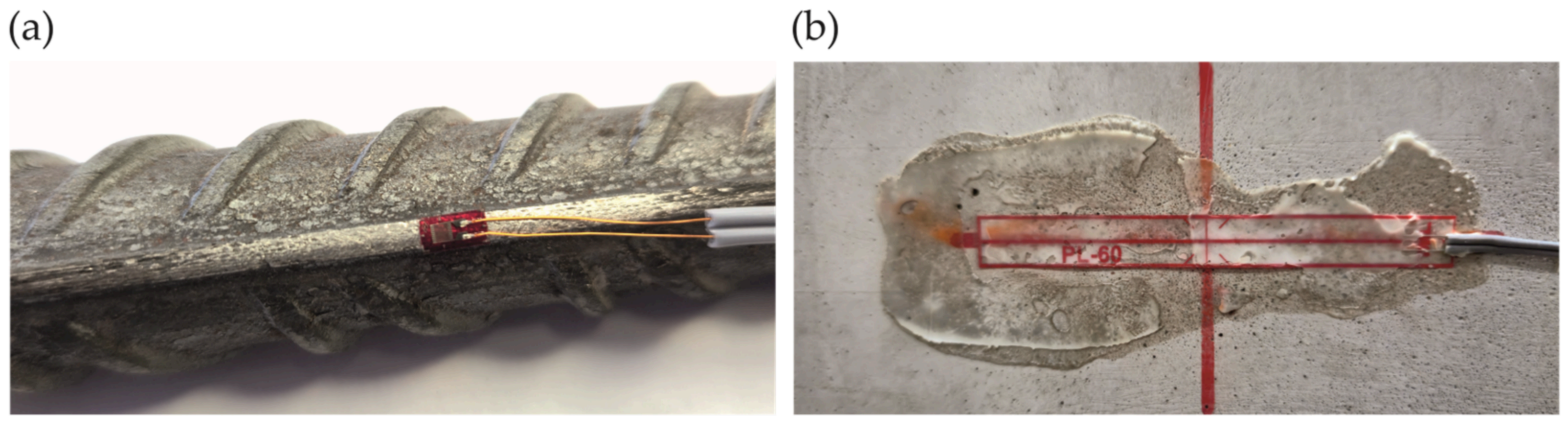
(**a**) A strain gauge (SG) on the reinforcement with a measuring length of 1 mm. (**b**) An SG on the concrete surface with a measuring length of 60 mm.

For many years, strain gauges, displacement transducers, and hydrostatic leveling systems have been employed in both research and practice [23]. Strain gauges (SGs) are utilized for the precise measurement of local strains. However, the typical measuring length of SG is only between 1 and 120 mm (Figure 2) [24], which means that SGs are limited in their suitability for monitoring the shear behavior of bridges. The displacement of a structure or the relative movement between two different structures can be precisely recorded using displacement transducers (DTs). In contrast to SGs, DTs can also measure larger deformations over longer distances [25]. However, since only the total deformation between two measuring points is recorded, it is not possible to precisely measure cracks or variations in crack widths. For this reason, DTs are only marginally suitable for monitoring the shear behavior of bridges. Hydrostatic leveling systems are geodetic measuring systems that have been utilized for the continuous monitoring of vertical deformations with a high degree of accuracy [26]. They provide valuable information about changes in the load-bearing behavior of a structure and are therefore often part of a comprehensive monitoring concept. However, it is not possible to directly monitor the shear behavior with hydrostatic leveling systems.

### 2.3. Fiber Optic Sensors

Fiber optic sensors are capable of measuring changes in strain and temperature over mid- to very-long measurement distances. The measurement setup consists of a glass-fiber sensor and an interrogator serving as the measuring unit. The interrogator injects laser light into the fiber, which is subsequently backscattered along the fiber by the fiber material itself. Depending on the deformation state of the fiber, the properties of the backscattered light change, which can be used to determine the strain changes along the fiber. The resulting backscatter pattern, also known as the optical fingerprint, is location-dependent, stable, and clearly reproducible [27].

Changes in fiber strain can be determined by evaluating the Rayleigh component of the backscattered laser light. This is achieved by comparing the local Rayleigh pattern with the reference state using a Fourier transformation, which enables strain to be determined with high spatial resolution at each point of the sensor fiber (measurement point spacing, e.g., 0.65 mm). Due to this quasi-continuous measurement, the method is also known as distributed fiber optic sensing (DFOS) [28].

**Figure 3:**
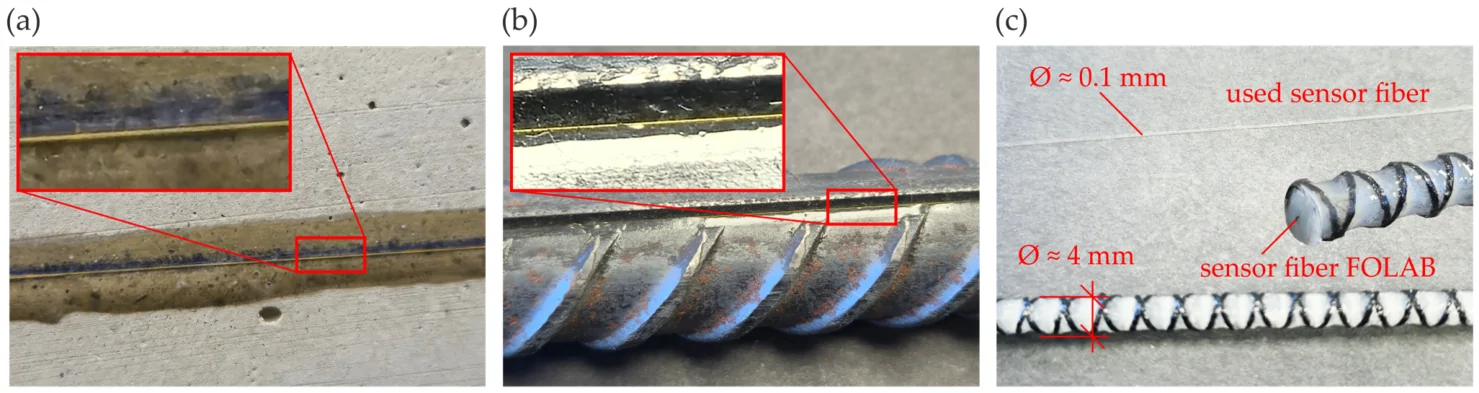
Installation of the fiber optic sensor (FOS): (**a**) directly on the concrete surface or (**b**) directly on the reinforcing bar. (**c**) Comparison of a polyimide-coated fiber with a fiber designed for use on construction sites, featuring an additional coating for integration into the structure.

The ability to measure strains almost continuously over the entire measurement length is a significant advantage of FOSs compared to SGs and DTs. This facilitates large-area monitoring and the early detection of structural changes, such as the formation or growth of cracks. Furthermore, FOSs exhibit resistance to electromagnetic interference and corrosion, and their low weight and diameter make them easy to integrate into or apply to existing structures [29,30]. FOSs can therefore be used for many different purposes in the construction industry [31,32]. This is substantiated by various studies on strain measurement in concrete, dynamic stress measurement, and, among other things, monitoring wood–concrete composite components [33,34,35,36,37,38]. In concrete structures, FOS can be installed directly on the concrete surface or on a reinforcing bar or—with an additional protective coating—embedded directly in the structure (Figure 3).

In the context of bridge monitoring, the exact location of critical shear cracks is typically unknown. FOSs overcome this limitation by providing quasi-distributed strain measurements over long distances, enabling simultaneous detection of local strain changes. This capability allows for the early identification of crack initiation and load redistribution, yielding direct insights into the load-bearing behavior of the structure.

The primary disadvantage of FOSs is that the sensor fiber is susceptible to breakage even at small crack widths. In the event of sensor fiber breakage at any point, no further measurements can typically be recorded along its entire length. The probability of the sensor fiber breaking within the area of a crack is mainly influenced by the adhesive used. In recent years, a significant number of studies have therefore been conducted on the installation of FOSs (e.g., [39,40,41]). Our investigations have shown that using an adhesive with a tailored elasticity allows local stress concentrations around a crack to be distributed over a defined sensor length, thereby preventing premature sensor fiber breakage at small crack widths. However, the potential for fiber breakage still exists at large crack widths. For this reason, it is advisable to avoid the placement of a sensor fiber in the tension zone of a structure or in areas where large cracks are anticipated, since the risk of sensor failure is significantly higher in these locations.

## 3. Experimental Test Setup

### 3.1. Test Specimens

To investigate the possibilities of using FOSs for monitoring the shear behavior of concrete bridges in more detail, six tests were carried out on reinforced concrete beams with different shear reinforcement ratios. All six beams had a rectangular cross-section with dimensions of h=0.34 m in height, b=0.20 m in width, and l=2.48 m in length. Furthermore, all six beams had identical longitudinal reinforcement of three Ø20 bars and Ø8 stirrups at 6 cm spacing outside the supports. Between the supports, the stirrup reinforcement exhibited variation. As a reference, the minimum shear reinforcement ratio was chosen in accordance with Eurocode 2 [42] and the National Annex for Germany [43], defined as follows:

(1) $$\rho_{w,min}=0.16\cdot \frac{f_{ctm}}{f_{ywk}}$$

In this context, $f_{ctm}$ denotes the mean axial tensile strength of the concrete and $f_{ywk}$ represents the characteristic tensile strength of the stirrup reinforcement. For the reinforced concrete beams investigated, the minimum shear reinforcement ratio lies between $\rho_{w,min}\approx 0.8‰$ and $\rho_{w,min}\approx 1.1‰$.

**Figure 4:**
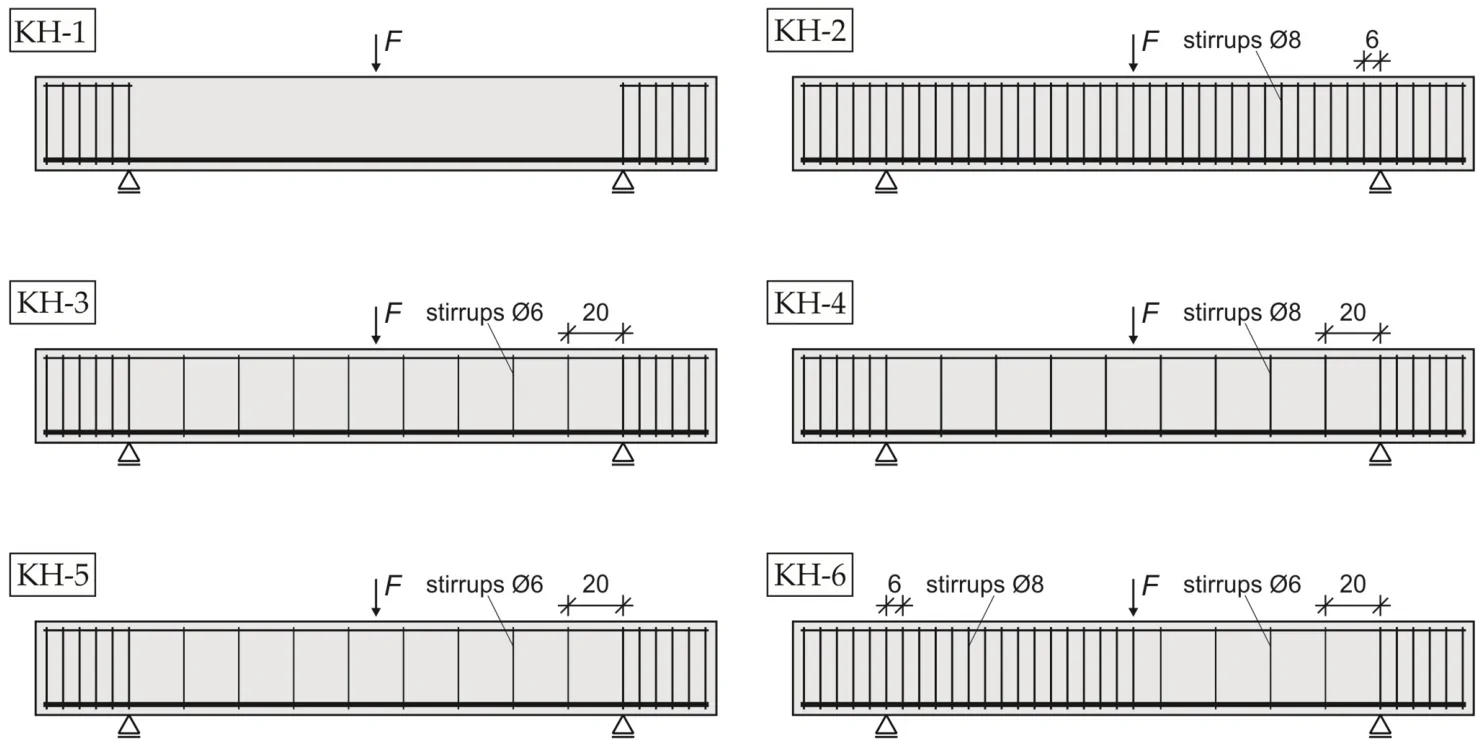
Overview of tested concrete beams KH-1 to KH-6 with different stirrup reinforcements.

In addition to a beam without shear reinforcement (KH-1), five beams with 1.3 to 8.1 times the minimum shear reinforcement ratio (KH-2 to KH-6) were examined. The variation was implemented to reflect the diversity of existing bridge infrastructure—although six tests cannot fully represent it—and to facilitate the evaluation of bridges without shear reinforcement as well as bridges with low shear reinforcement ratios. Figure 4 shows the reinforced concrete beams examined with the selected stirrup reinforcement. Test KH-6 is special in that the two shear regions (left and right) were reinforced differently in order to force failure on one side of the beam (monitoring region). For this purpose, the monitoring region (right) was reinforced with stirrup reinforcement of Ø6/20 (1.3 times the minimum shear reinforcement ratio) and on the other side (left)—where no failure was to occur—stirrup reinforcement of Ø8/6 (7.3 times the minimum shear reinforcement ratio) was installed. Further details on the stirrup diameters and spacings utilized, as well as the shear reinforcement ratios, are provided in Table 1.

The reinforced concrete beams were cast from normal-strength concrete with a maximum aggregate size of 16 mm. The mixture was designed to achieve concrete strength class C45/55. The mix proportions are shown in Table 2.

The test specimens were cast in coated wooden formwork and the concrete was compacted using external vibrators. According to [42], it can therefore be assumed that the lower longitudinal reinforcement and the stirrup reinforcement provide good bonding conditions. Subsequent to the concreting process, the upper surfaces of the test specimens were treated with a curing fluid, then removed from the formwork after seven days and stored in the test hall at a constant room temperature until testing.

To ascertain the material properties for all tests, 12 reference specimens (9 cylinders with Ø/h=15 cm/30 cm and 3 cubes with 15 cm edge length) were produced during each concreting process. After 28 days, the reference specimens were used to determine the compressive strength, splitting tensile strength, and modulus of elasticity of the concrete in accordance with EN 12390 [44,45,46]. The mean values of the concrete material properties are listed in Table 3.

B500B reinforcing steel was used for the lower longitudinal reinforcement (Ø20), and B500A reinforcing steel was used for the stirrup reinforcement (Ø6 and Ø8). The corresponding material properties were determined in accordance with EN ISO 15630-1 [47] using tensile tests. The results are displayed in Table 4.

### 3.2. Test Setup and Execution

**Figure 5:**
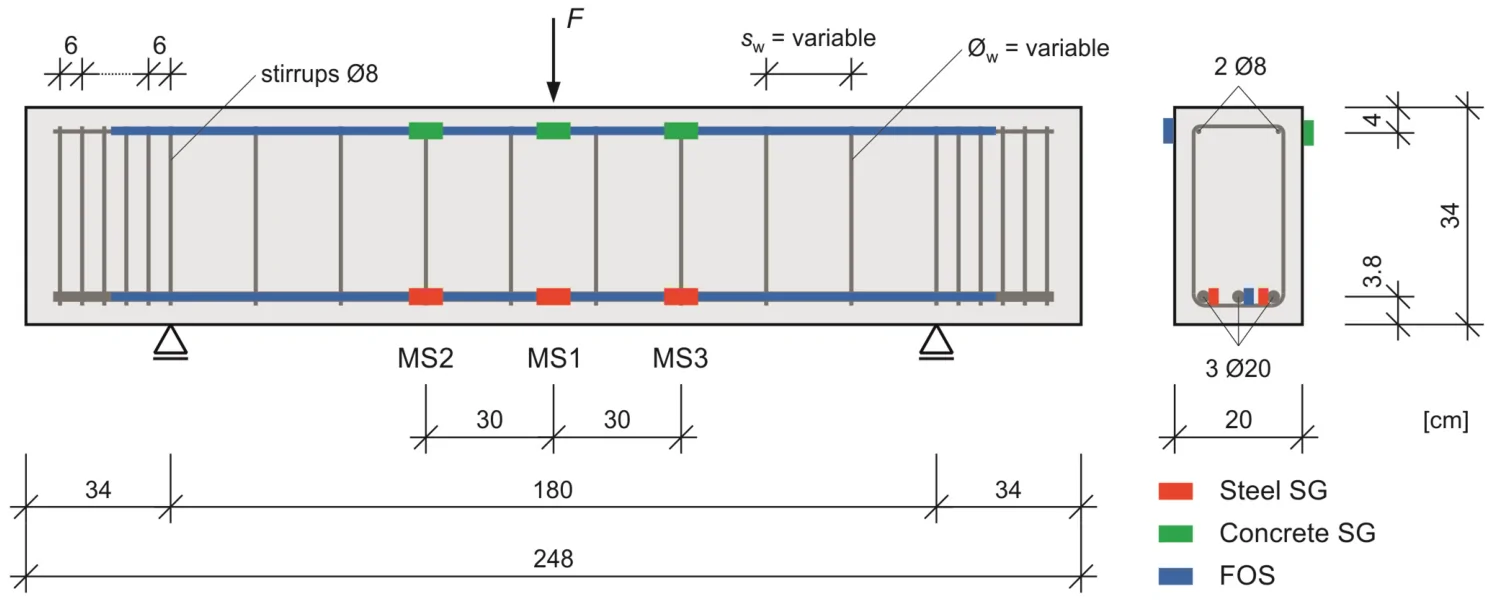
A schematic representation of the test setup with the positions of the SG and the FOS (not to scale).

The reinforced concrete beams were subjected to three-point bending tests under monotonically increasing loads until failure. The load was applied by a concentrated load in the middle of the beam ($a_{v}=180\;cm/2=90\;cm)$, avoiding any loads near supports ($a_{v}>2.0\cdot d=2.0\cdot 30.2\;cm=60.4\;cm$, according to [42]). Figure 5 illustrates the test setup.

The tests were conducted 28 days after casting. Load was applied using a hydraulic test cylinder and increased continuously until the reinforced concrete beam failed. After failure, the beams were unloaded and the crack patterns were traced, photographed, and digitized.

### 3.3. Instrumentation

#### 3.3.1. Overview of the Measurement Setup

As previously outlined in Section 1, two different configurations of FOSs were examined in this study. One FOS was installed on the lower longitudinal reinforcement and the other was placed on the concrete surface in the compression zone of the beam. The risk of failure of the FOS in the compression zone is significantly lower than that of the FOS in the tension zone, as cracking in the compression zone is not expected under service loads. However, it is unclear whether any cracks that occur will lead to strain concentrations in the compression zone and whether these can be measured with the FOS installed. If this proves to be the case, inferences regarding crack formation in the tension zone could be derived, thereby establishing the FOS in the compression zone as a suitable alternative to the vulnerable FOS in the tension zone. All measurements were carried out with a LUNA ODiSI 6104 interrogator (Luna Innovations Incorporated, Blacksburg, VA, USA) employing optical frequency domain reflectometry (OFDR) and a polyimide-coated fiber, sampling at 1 Hz. Additional specifications are detailed in the manufacturer’s datasheet [48]. To validate the measurement data, SGs were also installed on the reinforcement and on the concrete surface, and a DT was positioned at midspan beneath the beam. Figure 5 shows the positions of the SGs and FOSs.

#### 3.3.2. Fiber Optic Sensor in Tension Zone

In the tension zone, the FOS was installed on the middle bar of the lower longitudinal reinforcement. To ensure optimal adhesion of the FOS or adhesive to the reinforcement, loose particles on the surface of the reinforcing bar were removed by light sandblasting prior to installing the FOS. The FOS was then installed directly onto the surface of the reinforcing bar in the recess next to the longitudinal rib (see Figure 3b). The longitudinal rib typically extends without twists over the entire length of the reinforcement, offering the advantage that the FOS is better protected against mechanical impacts during installation and casting.

The UV-curing adhesive used was Anycubic UV Sensitive Resin Basic (Shenzhen Anycubic Technology Co., Ltd., Shenzhen, China), which offers two key advantages. On one hand, it can be applied quickly and precisely to different surfaces, as both the curing time and the curing speed can be influenced by the UV light. On the other hand, the UV-curing adhesive has a certain elasticity, so that local stress concentration in the area of a crack can be distributed over a certain length, which generally prevents the FOS from tearing in the case of small crack widths. To ensure optimal sensor alignment, the FOS was initially only glued to the reinforcing bar at specific points. In a subsequent step, the FOS was glued to the reinforcing bar along its entire length and sealed with an additional layer of adhesive for protection. To examine the gluing process and its possible effects on the FOS, a measurement was carried out during installation. The adhesive cured without significant heat generation, so no notable irregularities were detected in the measured values. Consequently, it can be assumed that the installation process was free of disturbances.

Prior to casting the beam, the characteristic points of the FOS were determined and stored in the interrogator for subsequent evaluation. These reference points included the start and end of the measurement and the midpoint of the beam. This ensures that—even after the beam has been cast—the data can still be clearly assigned, which is essential for evaluation. The measurement point spacing was set to 0.65 mm (see Section 2.3) to ensure the highest possible accuracy. In test KH-1, the FOS was damaged during casting, which means that no measurement data from the FOS in the tension zone are available for this specimen.

#### 3.3.3. Fiber Optic Sensor in Compression Zone

**Figure 6:**
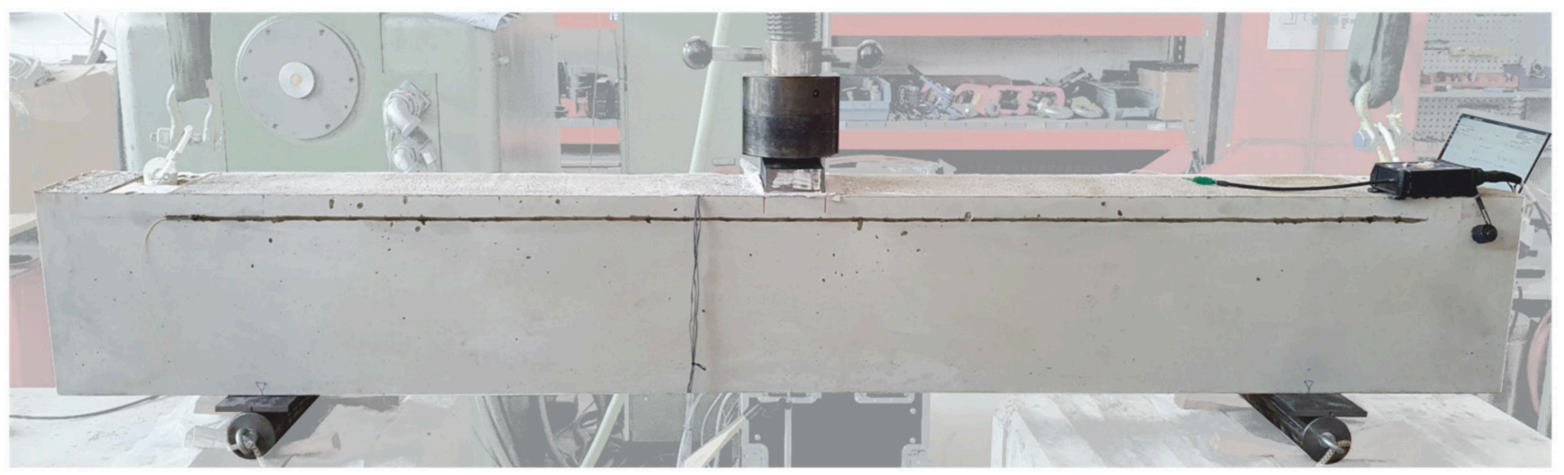
A reinforced concrete beam with an FOS installed in the compression zone (KH-1).

In the compression zone of the beam, the FOS was installed directly on the concrete surface in the area between the supports, 4 cm below the upper edge of the beam (see Figure 6). The UV-curing adhesive used for this purpose was the same as that used to install the FOS on the reinforcement. To achieve a line as straight as possible without sagging, the FOS was connected to the interrogator, pre-stretched to an almost constant strain of 0.2‰, and then glued to the concrete surface. Finally, an additional layer of adhesive was applied as protection. As it is currently unclear how the UV adhesive will behave on existing structures due to possible aging and creep effects under permanent strain of the FOS, a strain of only 0.2‰ was selected for the tests, which is not adjusted to the maximum possible concrete strain in the compression zone. The objective was also to investigate whether even a minor pre-strain would enable reliable measurement data, and thereby facilitate conclusions regarding the load-bearing capacity and crack behavior of reinforced concrete beams. To ensure that the applied strain did not affect the measurement data, the values were reset to zero immediately before the test. However, to guarantee traceability, the actual values were also recorded and stored. A comparison of the pre-strain values logged at installation and immediately before testing revealed no change, confirming negligible adhesive relaxation over this brief interval. Similar to the FOS in the tension zone, the characteristic points for the FOS in the compression zone were determined and stored for subsequent analysis. The measurement point spacing was also set to 0.65 mm. For tests KH-5 and KH-6, no complete measurements from the FOS in the compression zone are available due to an interrogator measurement error.

The FOSs in the compression zone were utilized to investigate whether conclusions regarding the load-bearing behavior could be drawn from the measured strains, and whether an FOS in the compression zone could be a viable alternative to an FOS in the tension zone, as the probability of failure is significantly lower (see Section 1). The two fundamental questions are as follows:

- Is it possible to traceably assign the detected strain concentrations to the cracks that have occurred in the tension zone?
- Can changes in load-bearing behavior be detected reliably?

#### 3.3.4. Strain Gauges and Displacement Transducers

To assess the global load-bearing and deformation behavior, the applied load was measured during the tests using a load cell located beneath the loading cylinder, while the deflection at midspan was recorded using a DT mounted beneath the beam. Additionally, to validate the FOS measurements at a later stage, SGs were installed on both the concrete surface and the longitudinal reinforcement in three measurement sections (MS1 to MS3), located at midspan and 30 cm off center toward the supports (see Figure 5, green and red markers).

The validation of the measured values was achieved by installing six SGs (each with a measuring length of 1 mm, see Figure 2a) on the outer two bars of the lower longitudinal reinforcement (three SGs per bar) in the tension zone. In the compression zone, a total of three SGs were installed on the concrete surface at the same height as the FOS. Due to the non-homogeneous behavior of concrete, SGs with a measuring length of 60 mm were selected for this purpose (see Figure 2b). The concrete SGs were installed on the side opposite the FOS, as the measurement systems could not be placed on top of each other and, secondly, installing the SGs with the required adhesive causes a brief, locally limited heat buildup that could have damaged the FOS.

## 4. Experimental Results and Discussion

### 4.1. Failure Modes

**Figure 7:**
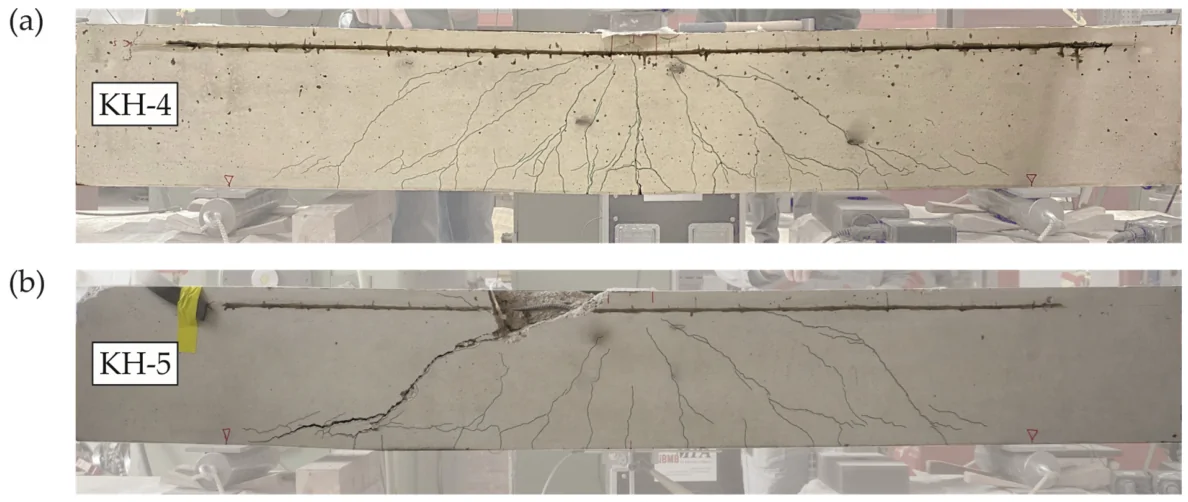
Concrete beams immediately after testing, with cracks marked: (**a**) KH-4 showing flexural failure; (**b**) KH-5 showing shear failure.

In test KH-1 (without shear reinforcement) and in tests KH-3, KH-5, and KH-6 (with 1.5, 1.8, and 1.3 times the minimum shear reinforcement ratio, respectively), brittle shear failure occurred in the form of a critical shear crack (KH-1) or a critical shear crack accompanied by failure of the stirrup reinforcement (KH-3, KH-5, and KH-6). In contrast, tests KH-2 and KH-4, conducted with higher shear reinforcement ratios of 8.1 and 2.3 times the minimum shear reinforcement, respectively, exhibited ductile flexural failure characterized by yielding of the longitudinal reinforcement at the critical flexural crack. Figure 7 shows beam KH-4 after flexural failure and beam KH-5 after shear failure.

When the flexural reinforcement in shear tests is not sufficiently overdesigned, flexural failure becomes more likely. Shear cracks then weaken the flexural capacity of the beam, causing it to fail in flexure before shear. Since the longitudinal reinforcement in bridges is typically not sufficiently overdesigned, effects similar to those observed in tests KH-2 and KH-4 can also occur in bridges. Although the monitoring of flexural behavior was not the primary focus of the investigation, these two tests also provide valuable information for further evaluation and insight into the influence of the shear reinforcement ratio on the failure mode of reinforced concrete beams.

**Figure 8:**
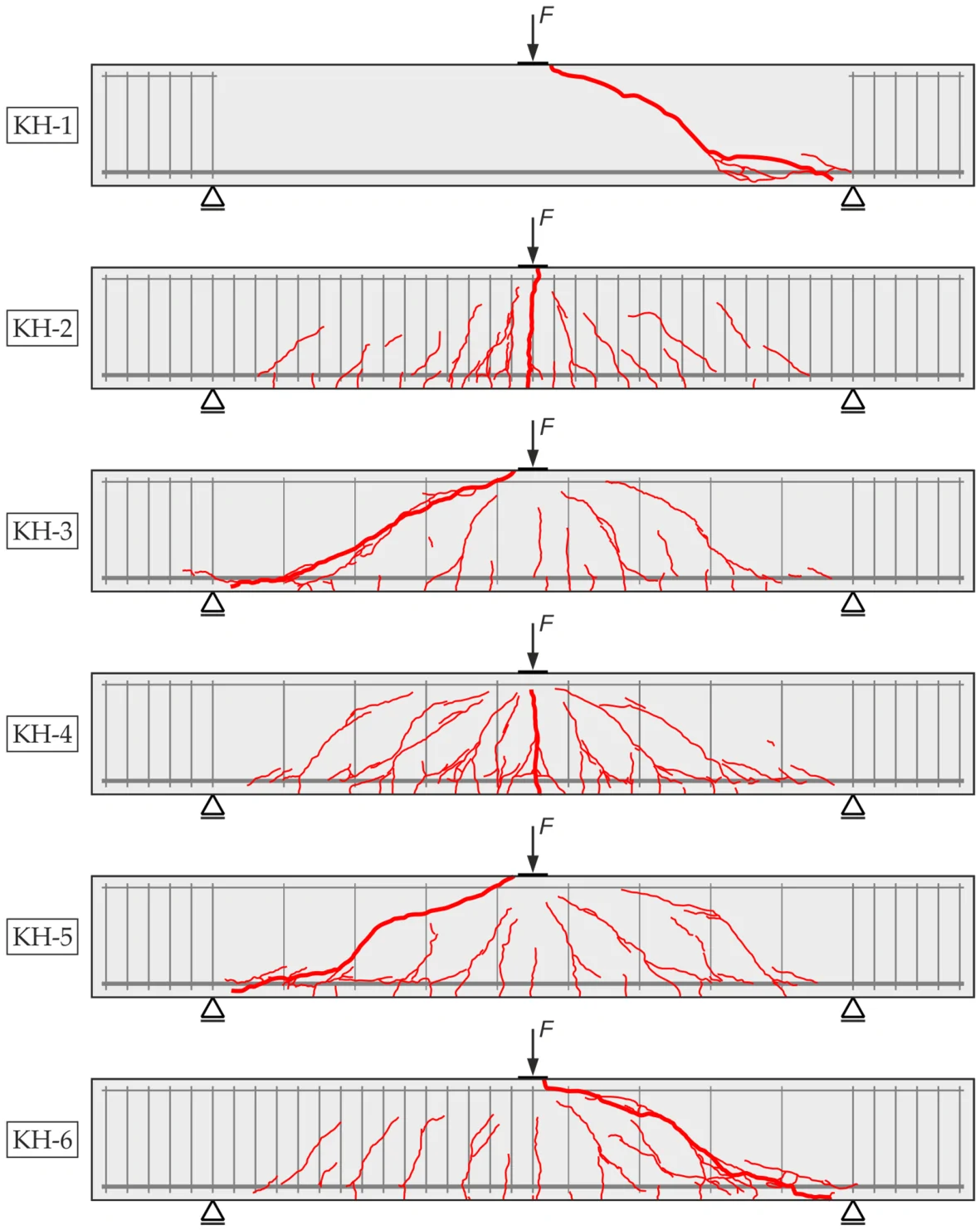
Crack patterns of beams KH-1 through KH-6 immediately after testing.

Figure 8 shows the graphically recorded and digitized crack patterns from all tests. The critical shear cracks (KH-1, KH-3, KH-5, and KH-6) and the critical flexural cracks (KH-2 and KH-4) are highlighted with thicker crack lines.

**Figure 9:**
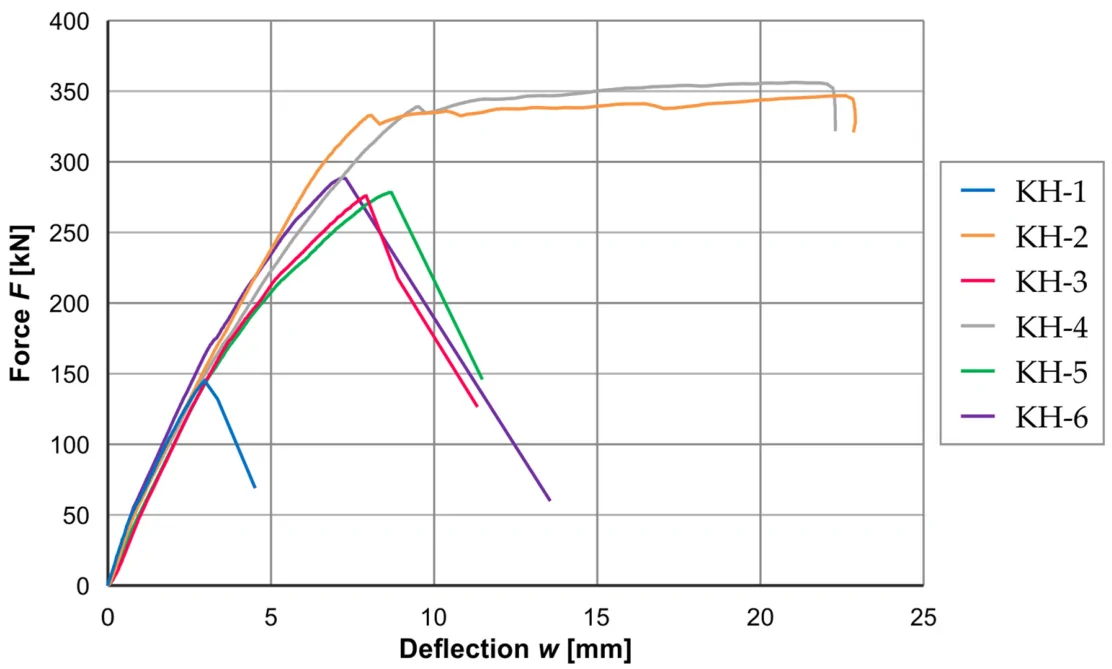
Force–deflection curves for tests KH-1 to KH-6.

Figure 9 shows the corresponding force–deflection curves for all tests. Tests KH-1, KH-3, KH-5, and KH-6 display brittle shear failure, evident as an abrupt drop in the curve. In contrast, tests KH-2 and KH-4 exhibit ductile flexural failure due to yielding of the longitudinal reinforcement at midspan, manifesting as a plateau in the curve. Both tests were terminated prematurely once a maximum deflection of approximately 22 mm was reached.

The failure loads and corresponding failure modes are listed in Table 5. To facilitate classification, the shear reinforcement ratio normalized to the minimum ($\rho_{w}/\rho_{w,min}$) is also provided.

### 4.2. Analysis of Measurement Data

#### 4.2.1. Overview of the Evaluation Approach

The evaluation of the FOS focuses on tests KH-1 (without shear reinforcement) and KH-3 (1.5 times the minimum shear reinforcement ratio), which failed in shear, and on test KH-2, which failed in flexure. The load stages considered in the analysis are as follows: 0% (blue line), approximately 30% (orange line), approximately 80% (purple line), and 100% (gray line) of the measured failure load $F_{u}$. While 0% and 100% define the unloaded state and the failure load level, the intermediate stages are intended to represent a possible service load level and a design load level rather than being interpreted as fixed normative levels. Consequently, these stages can serve as examples for the monitoring and evaluation of bridges. Since the remaining tests (KH-4, KH-5, and KH-6) yielded similar results, they are presented only in Appendix A and are not discussed separately in this article. In test KH-1, however, damage to the FOS on the longitudinal reinforcing bar during casting (see Section 3.3.2) confined the evaluation to the measurement data from the compression zone.

To facilitate further analysis and interpretation of the measurement data, the respective calculated strain profiles under idealized conditions were determined for FOSs in both the tension zone and the compression zone.

Under the assumption that the beam is in cracked state II and the reinforcement behaves as a linearly elastic material, the strain in the lower longitudinal reinforcement can be determined by an equilibrium analysis. The derivation of this equation is as follows:

(2) $$\varepsilon_{s}=\frac{F\cdot l_{eff}}{4\cdot z\cdot A_{sl}\cdot E_{sm}}$$

**Figure 10:**
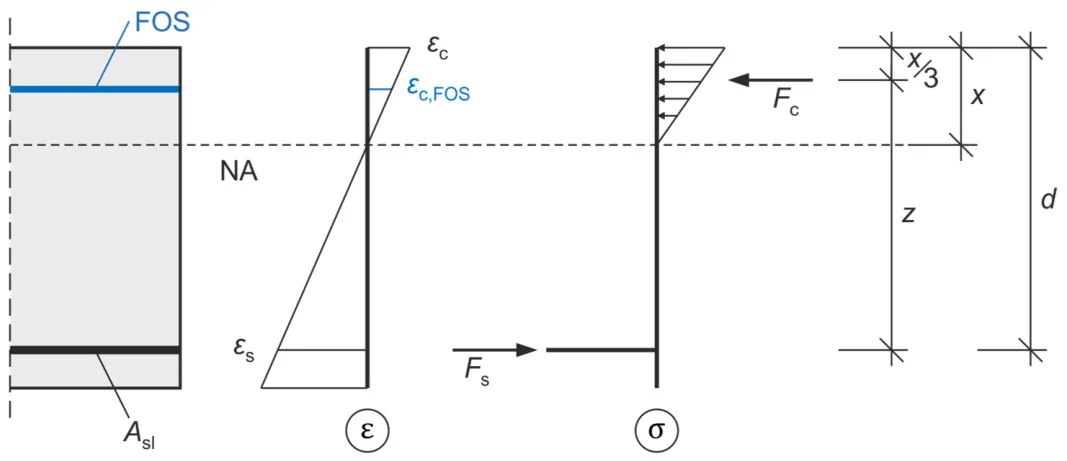
Strain (ε) and stress (σ) assumptions for calculating the strain at the height of the FOS under idealized conditions.

**Figure 11:**
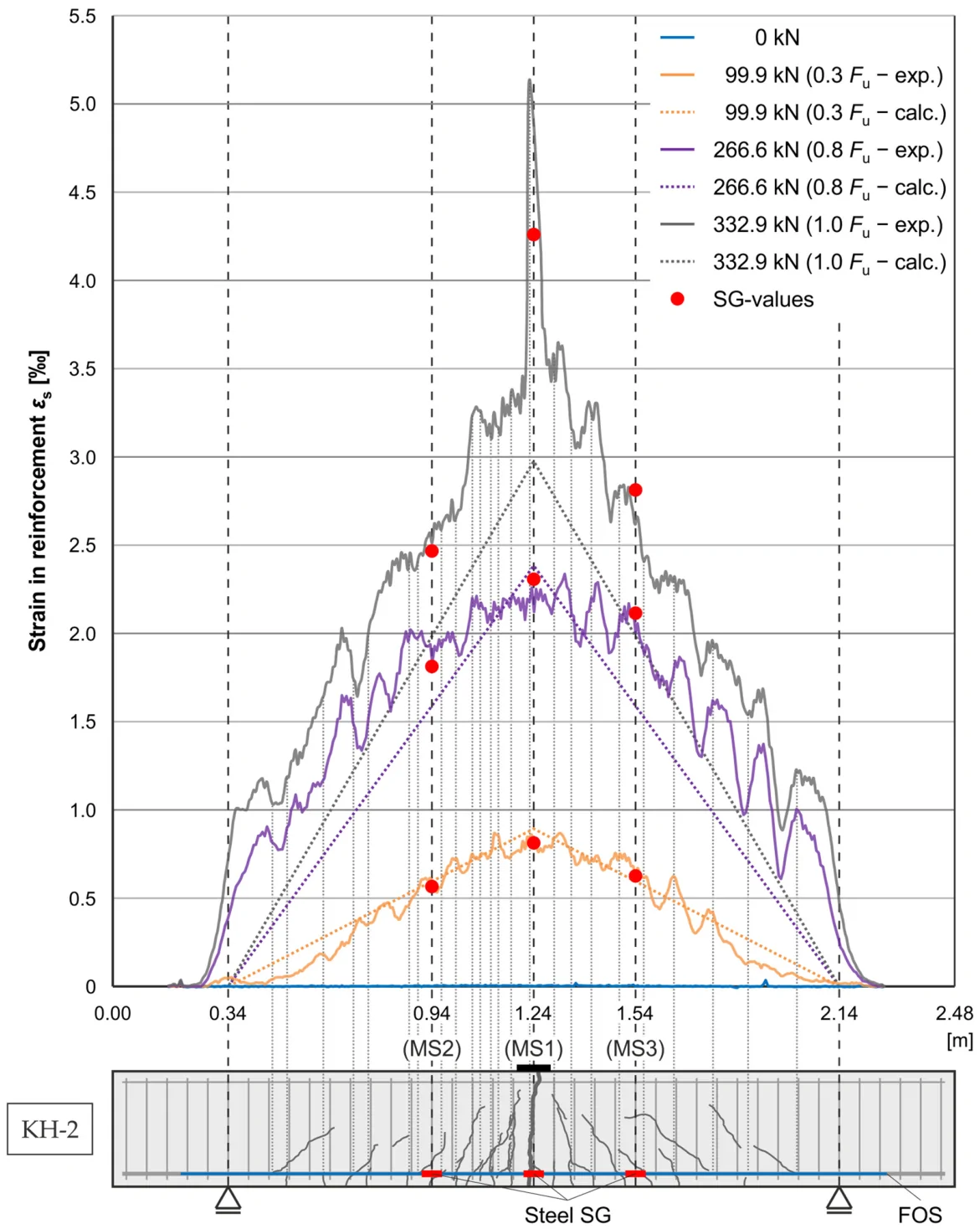
Evaluation of the measured values determined using an FOS and SGs on the lower longitudinal reinforcement in the tension zone of beam KH-2 with flexural failure.

**Figure 12:**
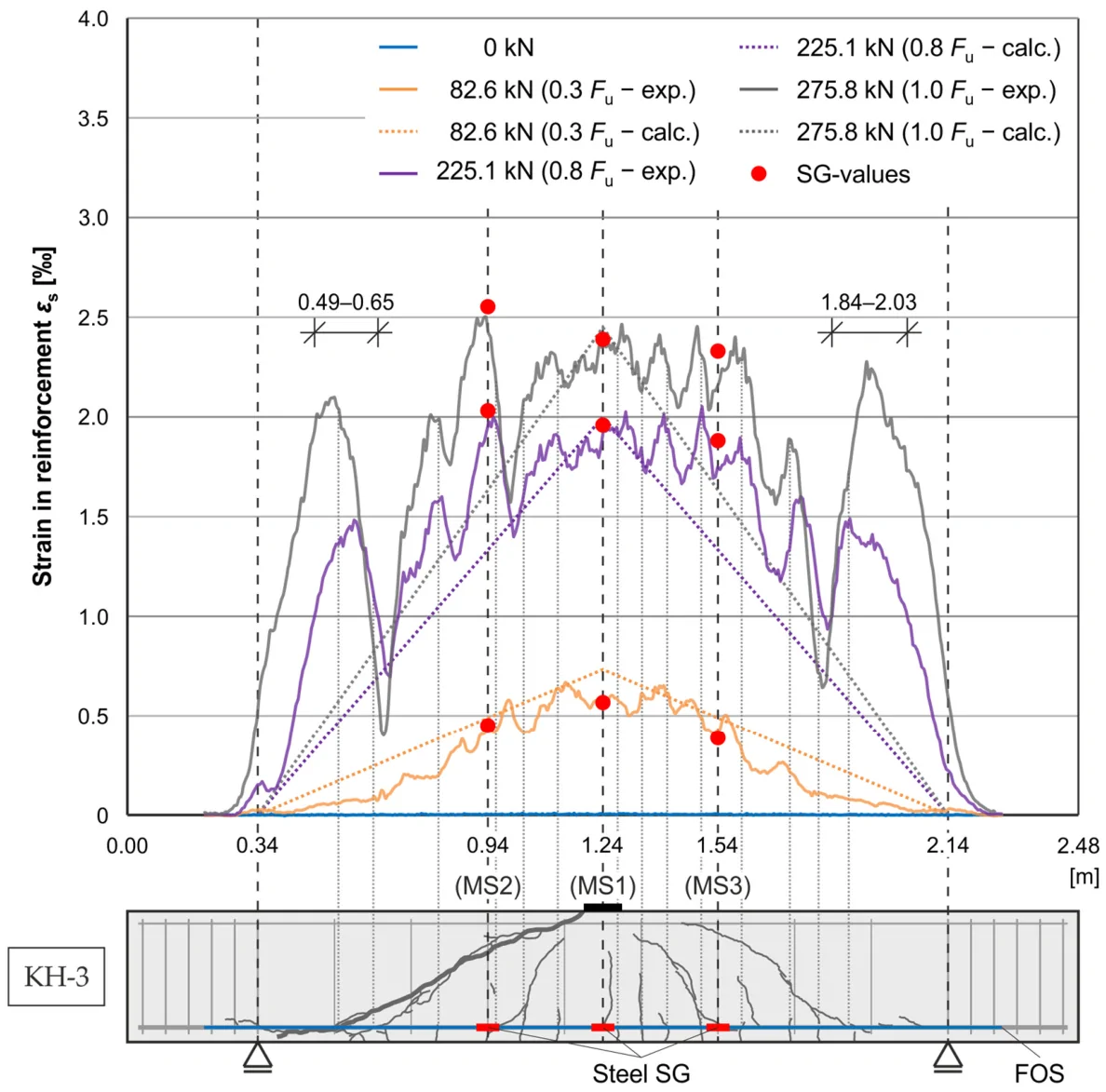
Evaluation of the measured values determined using an FOS and SGs on the lower longitudinal reinforcement in the tension zone of beam KH-3 with shear failure.

In this equation, variable F denotes the applied force, $l_{eff}$ is the effective length of the beam, $A_{sl}$ is the cross-sectional area of the longitudinal reinforcement, $E_{sm}$ is the mean modulus of elasticity of the reinforcement, and z is the inner lever arm (see Figure 10). The calculated strain profiles are shown in Figure 11 and Figure 12 as orange, purple, and gray dotted lines (labeled “calc.”).

The calculated strain profile under idealized conditions for the FOS in the compression zone was derived from the following assumptions:

- Linear strain distribution across the section height;
- Concrete and reinforcing steel behave in a linearly elastic manner;
- Cross-section is cracked in the tension zone;
- No tensile contribution from concrete.

By equating the concrete compressive force and the tensile force in the reinforcement (internal force equilibrium, see Figure 10), the following equation is obtained:

(3) $$E_{cm}\cdot b\cdot \frac{x}{2}\cdot \varepsilon_{c}=E_{sm}\cdot A_{sl}\cdot \varepsilon_{s}$$

In this equation, $E_{cm}$ denotes the mean modulus of elasticity of the concrete, b denotes the cross-sectional width, and x denotes the neutral-axis depth.

Assuming plane sections remain plane, the strain in the lower longitudinal reinforcement $\varepsilon_{s}$ is derived from the concrete strain $\varepsilon_{c}$ at the top fiber by similar triangles. Under these assumptions, the neutral-axis depth x remains constant, so the concrete strain cancels out and the expression reduces to a polynomial form. This results in Equation (4), where d denotes the effective depth of the cross-section.

(4) $$E_{cm}\cdot b\cdot \frac{x^{2}}{2}=E_{sm}\cdot A_{sl}\cdot \left(d-x\right)$$

The neutral-axis depth x can now be determined by standard algebraic methods. To determine the concrete strain $\varepsilon_{c}$, the bending-moment equilibrium about the top fiber of the beam (Equation (5)) is set up and solved for the corresponding bending moment M.

(5) $$M=E_{cm}\cdot b\cdot \varepsilon_{c}\cdot \frac{x^{2}}{6}+E_{sm}\cdot A_{sl}\cdot \varepsilon_{c}\cdot \left(1-\frac{d}{x}\right)\cdot d$$

Ultimately, the concrete strain at the height of the FOS $\varepsilon_{c,FOS}$ is obtained from Equation (6). This is based on a linear strain distribution and on the FOS located 4 cm below the top fiber of the beam (see Figure 5 and Section 3.3.3).

(6) $$\varepsilon_{c,FOS}=\varepsilon_{c}\cdot \left(1-\frac{0.04\;m}{x}\right)$$

**Figure 13:**
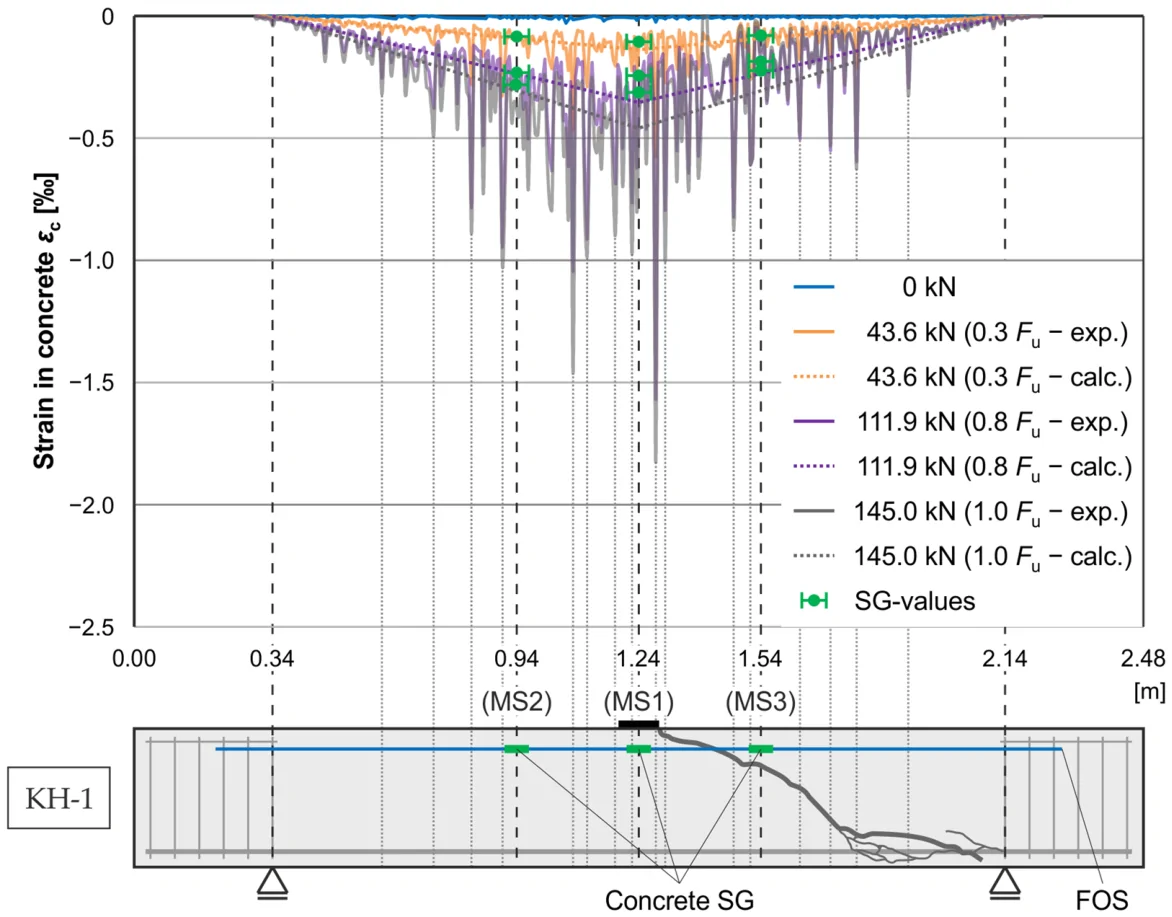
Evaluation of the measured values determined using an FOS and SGs on the concrete surface in the compression zone of beam KH-1 with shear failure.

**Figure 14:**
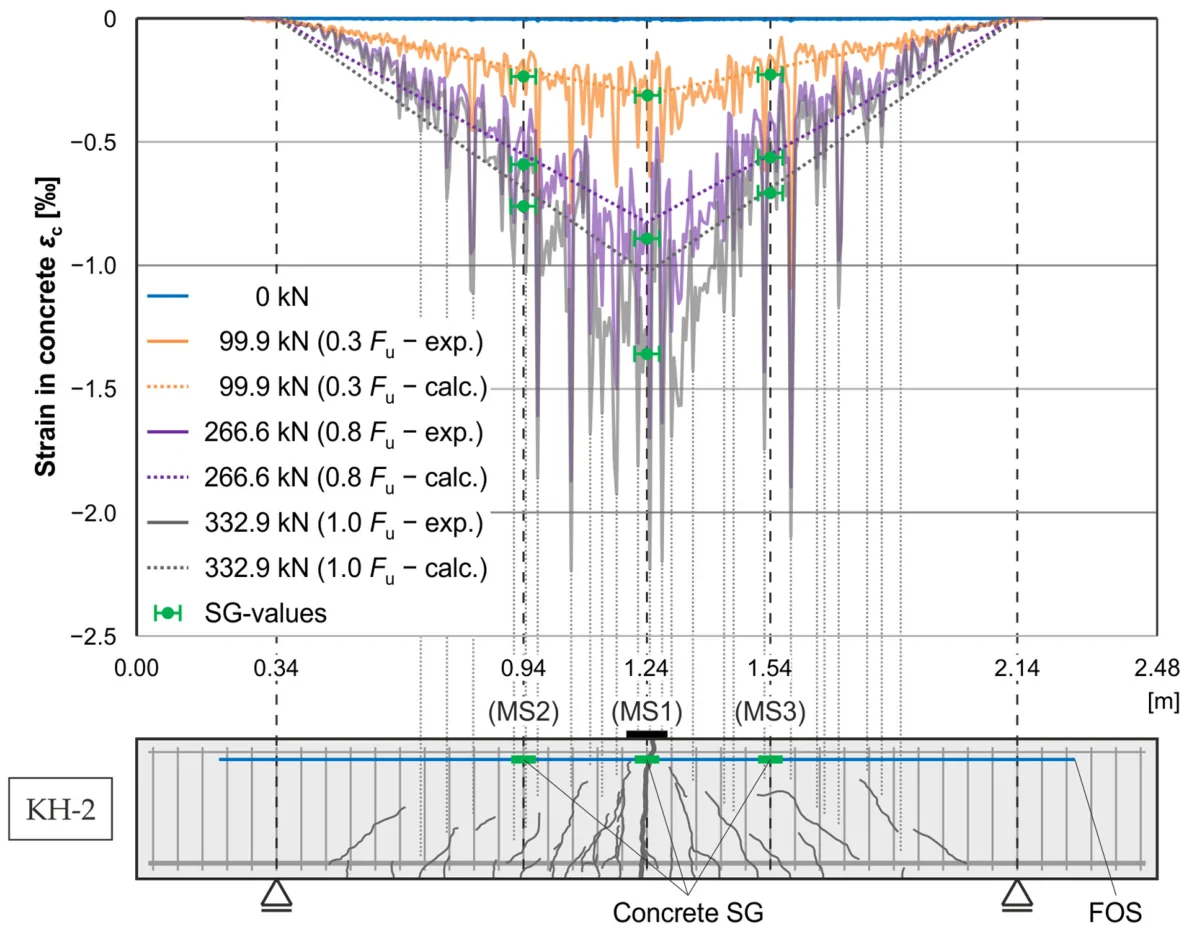
Evaluation of the measured values determined using an FOS and SGs on the concrete surface in the compression zone of beam KH-2 with flexural failure.

**Figure 15:**
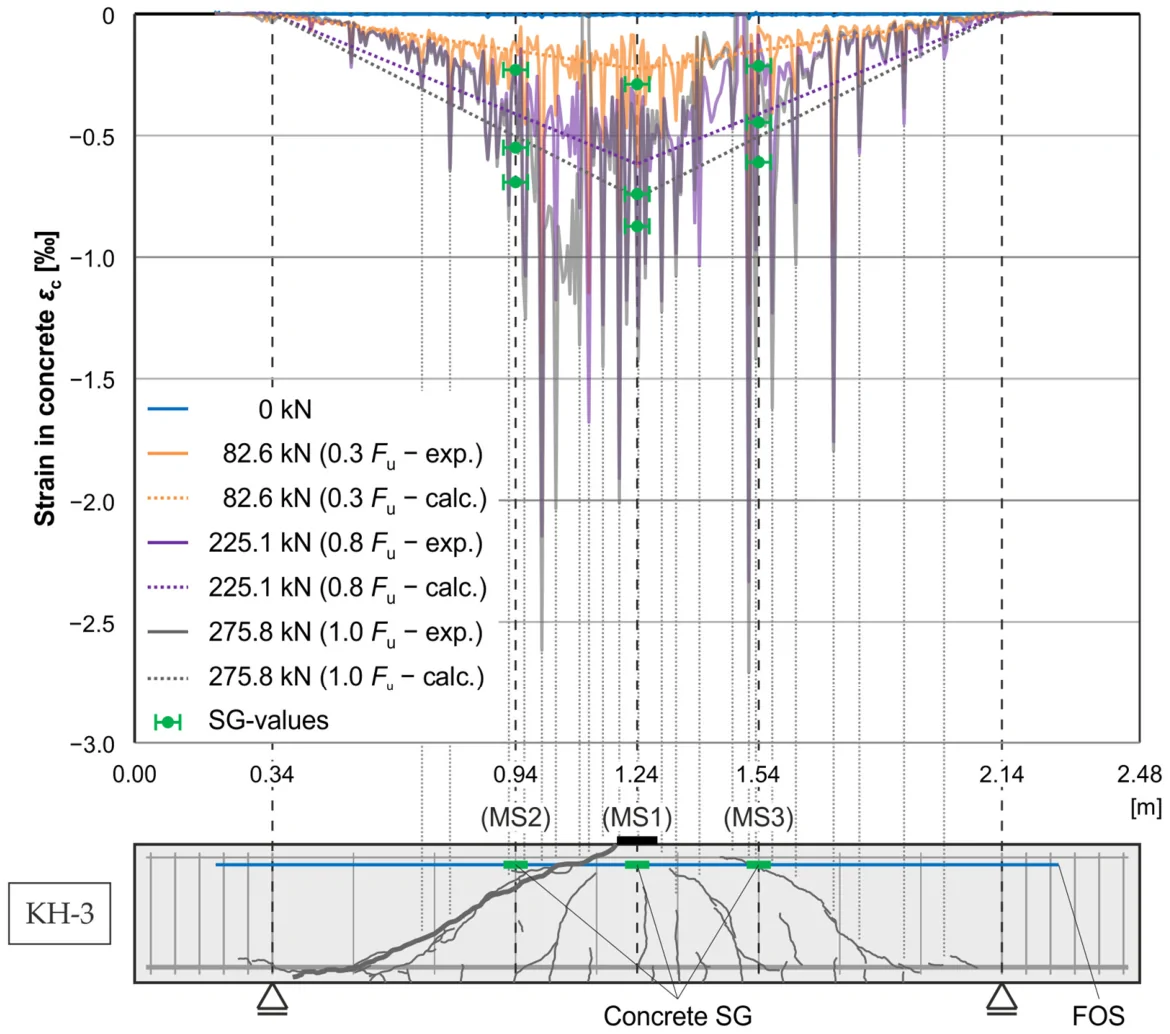
Evaluation of the measured values determined using an FOS and SGs on the concrete surface in the compression zone of beam KH-3 with shear failure.

The strain profiles calculated from the outlined assumptions are shown in Figure 13, Figure 14 and Figure 15 for the examined load levels as orange, purple, and gray dotted lines (labeled “calc.”).

#### 4.2.2. Strain Measurements in Tension Zone

The evaluation of the FOS in the tension zone is illustrated in Figure 11 for test KH-2 with flexural failure and in Figure 12 for test KH-3 with shear failure. In both figures, the solid blue, orange, purple, and gray lines (labeled “exp.”) indicate the load levels. Since the data exhibit only minimal variation with the selected measurement-point spacing of 0.65 mm—as confirmed by a sensitivity check between full resolution and reduced data—only every tenth measurement point is shown for clarity.

A comparison of tests KH-2 and KH-3 shows that at approximately 30% of the failure load ($0.3\;F_{u}$), the steel strain profiles are generally similar. However, at approximately 80% of the failure load ($0.8\;F_{u}$), significantly larger differences appear. These differences can be primarily attributed to the higher shear reinforcement ratio in test KH-2, enabling greater load redistribution. In test KH-3, this is particularly evident in the regions near the supports, from x=0.49 m−0.65 m and from x=1.84 m−2.03 m, where large strain concentrations can be observed. These occur at higher loads—approximately 60–70% of the failure load—indicating shear crack formation leading to brittle failure. With a further increase in load and an absence of redistribution options, strains may be experienced in these regions that are of an equivalent magnitude to the maximum strain at midspan. This phenomenon can be clearly observed at 100% of the failure load ($1.0\;F_{u}$). Furthermore, the strain in the surrounding area decreases significantly, indicating imminent failure. By contrast, test KH-2 exhibits an almost continuous increase in strain. Only in the area of maximum stress, where the yield strength of the reinforcement is exceeded, a significant increase can be detected and the imminent flexural failure of the beam can be identified.

Also shown in the diagrams in Figure 11 and Figure 12, strains measured by the steel SGs are marked with red dots, each dot representing the average of two SGs in the corresponding measurement section. Comparing the strains measured with the SGs (red dots) with those measured with the FOS (solid orange, purple, and gray lines) reveals a high degree of agreement. The small deviations can be attributed to the fact that the SGs were not located on the middle longitudinal reinforcing bar—as the FOS was—but rather on the two outer bars (see Figure 5). When the SG results (red dots) are compared with the calculated strain profiles (orange, purple, and gray dotted lines), the measured strains also demonstrate good agreement on average with the idealized strain profiles.

To provide a more detailed evaluation of the measurement data, Figure 11 and Figure 12 also show the reinforced concrete beams with the corresponding crack patterns. The locations at which a crack crosses the lower longitudinal reinforcement are indicated by a light-gray dotted line. A comparison of the measurement data from the FOS with the respective crack pattern reveals a substantial correspondence between the measured strain concentrations and the observed cracks. However, minor deviations are evident in some cases. These deviations can be attributed either to slight differences in the crack patterns within the beam or to possible load redistributions during the initiation of new cracks. It should also be noted that the crack patterns were traced manually on the beams and subsequently digitized (see Section 3.2), which may have introduced further minor deviations due to transfer errors. Furthermore, cracks that have closed again after unloading may be absent from the crack patterns. Nevertheless, the results show a generally high degree of consistency, confirming the suitability of FOSs in the tension zone for the early detection of cracks—usually significantly earlier than visual identification.

#### 4.2.3. Strain Measurements in Compression Zone

The evaluations of the FOS in the compression zone are shown in Figure 13, Figure 14 and Figure 15 for tests KH-1, KH-2, and KH-3, respectively. Because the concrete surface of the beam is inhomogeneous (aggregate, cement paste and possibly pores), the measurement data demonstrate locally higher scatter. To reduce this scatter, the figures plot values averaged over a 5.85 mm measurement distance (nine-point average). The smoothing window was chosen after a sensitivity analysis, revealing that a smaller window produced large fluctuations and a larger window led to excessive smoothing. Therefore, the nine-point average proved to be a good compromise for crack detection. Moreover, it should be noted for the evaluation of the measurement data that the measured strain in the compression zone is also influenced by other factors (e.g., bending, concrete nonlinearity, and load redistribution), which can complicate the interpretation of the strain profiles.

**Figure 16:**
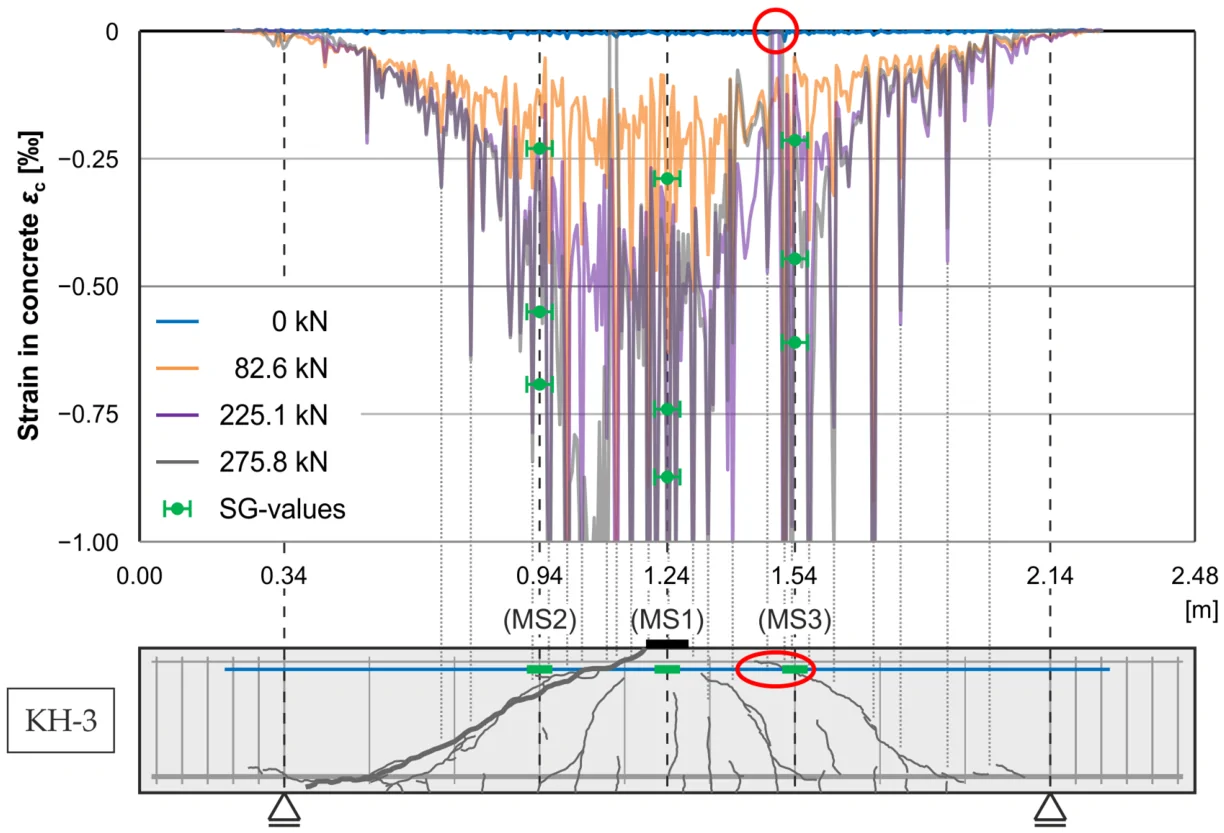
Evaluation of the measured values determined using an FOS and SGs on the concrete surface in the compression zone of beam KH-3 with shear failure for the strain range from 0‰ to −1‰.

First, it can be observed that the strain profiles of tests KH-1, KH-2, and KH-3 match the bending moment diagram and the expected behavior. Compared to measurements with the FOS on the lower longitudinal reinforcement, the measurements in the compression zone show significantly more local strain concentrations. To provide a more comprehensive overview, Figure 16 shows an enlarged section for the strain range from 0‰ to −1‰ for test KH-3.

A comparison of the strain profiles in Figure 13, Figure 14 and Figure 15 shows that, at approximately 30% of the failure load ($0.3\;F_{u}$) and approximately 80% of the failure load ($0.8\;F_{u}$), the profiles for KH-1, KH-2, and KH-3 are essentially the same with no discernible disparities. In test KH-1 (without shear reinforcement), strains are significantly lower than in tests KH-2 and KH-3 with shear reinforcement. This can be attributed to the fact that KH-1 exhibits a significantly lower load-bearing capacity due to the absence of shear reinforcement. A detailed analysis of the local strain concentrations reveals a strong correspondence between strain concentrations—particularly near supports—and the observed crack patterns. The formation of a crack, namely the transition from an uncracked to a cracked state, causes a pronounced strain increase in the compression zone, and FOSs reliably detect these changes. However, as cracks orient themselves with the loading direction under increasing load, this correlation is most evident during the crack initiation phase and at midspan, where cracks run almost vertically. With further load increase and no redistribution options, the cracks extend deeper into the compression zone. For the strain concentrations that have previously been detected, this results in an increase in the measured strains. However, no significant new strain concentrations emerge even at 100% of the failure load ($1.0\;F_{u}$).

**Figure 17:**
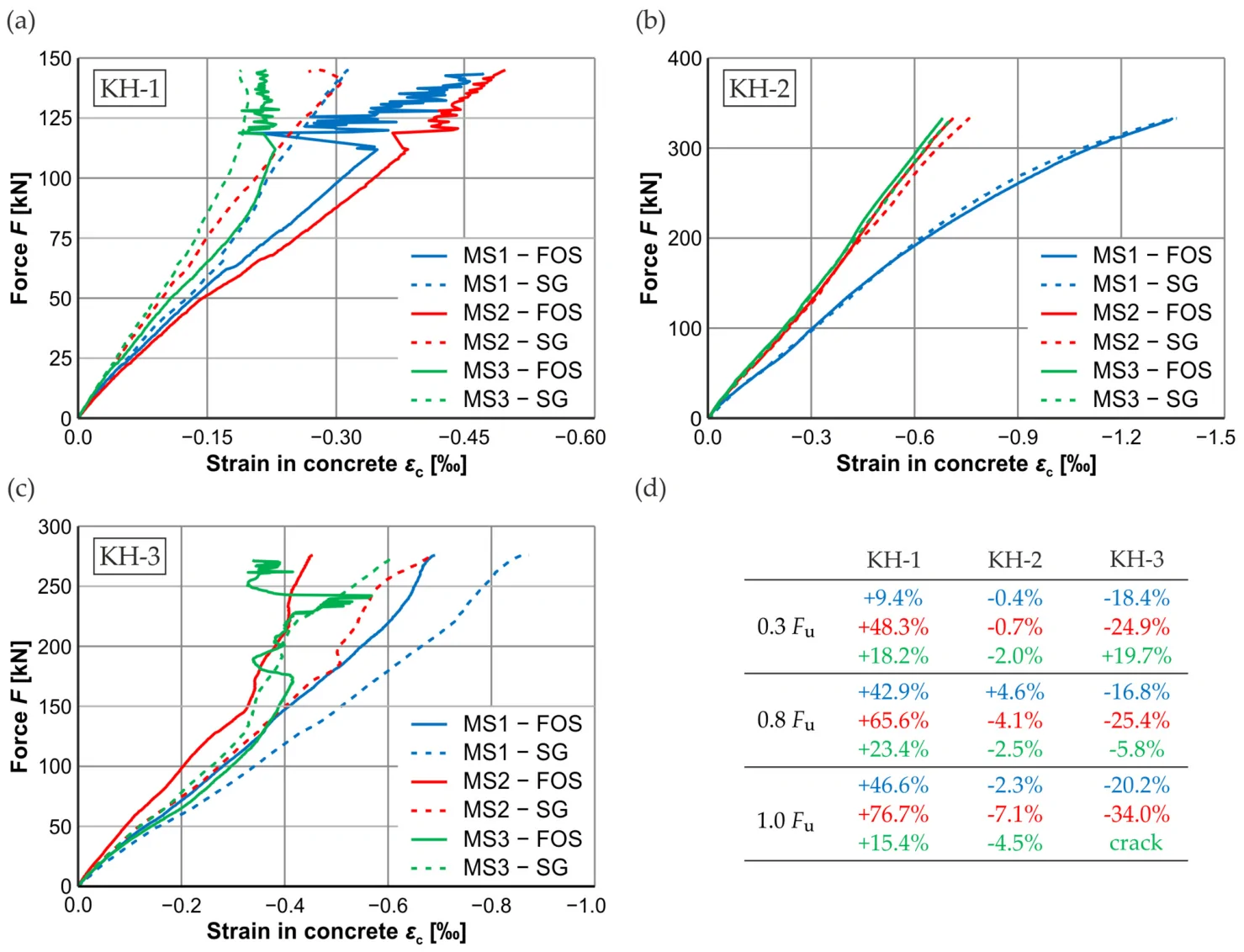
Comparison of strains measured using an FOS and SGs in the compression zone: (**a**) beam KH-1; (**b**) beam KH-2; (**c**) beam KH-3. (**d**) Deviations between FOS and SG measurements (MS1: blue; MS2: red; MS3: green).

A direct comparison between the strains measured with concrete SGs (measurement length: 60 mm) and the strains measured with FOSs (measurement point spacing: 0.65 mm; evaluation length: 5.85 mm) is not possible due to the disparity in sensing lengths. Therefore, an average strain from the FOS over 60 mm measurement length was determined at each location of the concrete SGs and compared to the measured values of the SGs. Figure 17 presents the force–strain curves for measurement sections MS1, MS2, and MS3 in tests KH-1, KH-2, and KH-3. To allow for a clearer assessment of their agreement, the quantitative deviations between FOSs and SGs at the selected load stages are also given. Overall, the strains exhibit a reasonable degree of agreement between FOSs and SGs. In test KH-2 with flexural failure, the two systems produce nearly identical curves. In tests KH-1 and KH-3 with shear failure, the overall shape of the strain curves is consistent, but pronounced deviations occur in some cases, which can be primarily attributed to the different sensor positions on opposite sides of the beam and to the inhomogeneity of the concrete (see Section 3.3.4). In test KH-3, measurement inaccuracies in the form of noise become evident in MS3 from approximately 60% of the failure load with significant deviations from approximately 85% of the failure load. This behavior results from a crack crossing the FOS in the area under consideration (see red circles in Figure 16). As a result, local tensile strains emerge, causing a decrease in the overall strain. Nevertheless, the agreement between the FOS and SGs remains satisfactory, and the SG measurements—plotted as green dots with local limitation—are shown in Figure 13, Figure 14, Figure 15 and Figure 16.

A comparison of the measured strains (green dots) with the calculated idealized strain profiles (orange, purple, and gray dotted lines) shows good overall agreement. However, since the reinforced concrete beams did not behave as a purely linear-elastic material during testing, the measured strains are slightly higher than the idealized values.

In conclusion, the FOS in the compression zone allows for the detection of cracks and significant changes in load-bearing behavior. In the tests, an assumed pre-strain of 0.2‰—chosen as a conservative, non-standard value well below the actual compressive strains observed in the compression zone—proved sufficient to deliver clear, reproducible signals. However, reliable failure prediction using an FOS in the compression zone proves largely infeasible, since increasing load neither generates new strain concentrations nor significantly increases previously identified strain concentrations. This limitation arises because cracks are typically inclined, so strain concentrations are not necessarily located directly above a crack. A distinctive effect appears when the neutral axis reaches or passes the FOS, allowing tensile strains to be detected. Figure 16 illustrates this in test KH-3 at approximately 1.49 m, as indicated by the red circles. The remaining tests, exhibiting flexural or shear failure (see Appendix A), also show similar behavior. However, because cracks generally reach the level of the FOS only immediately prior to failure, this indicator cannot serve as a criterion for monitoring reinforced concrete beams or bridges. Placing the FOS closer to the neutral axis would also be ineffective, as it would negate the advantage of the very low probability of FOS failure.

### 4.3. Assessment of the Results for Early Failure Detection

To ensure effective monitoring of bridges with deficiencies in shear capacity, the structure must exhibit adequate warning behavior prior to failure. According to [49], a warning is adequate only if the failure mode is ductile, the warning signal is clearly perceptible, and the time interval between detection and failure is long enough to mitigate damage.

A large number of studies on the shear capacity of concrete beams with and without shear reinforcement have been documented in the literature (for example [50,51,52]). The failure of reinforced concrete beams without shear reinforcement is typically characterized by brittle shear failure with little or no warning [53]. According to [54], studies have demonstrated that, with the assistance of new measurement systems, a certain degree of warning can be detected even in beams without shear reinforcement, and that beams can therefore fall between ductile and brittle failure modes. Current studies are investigating the potential of FOSs in detecting warning signs of failure in beams under shear load (e.g., [28]). The primary challenge in this context does not lie in the detection of shear cracks, but rather in the prediction of their behavior under increasing loads, and identification of the crack that will ultimately lead to shear failure of the beam.

Additionally, the measurement data obtained from the FOS can be used for further analysis. According to the European Standard, structural behavior is classified—depending on the shear reinforcement ratio $\rho_{w}$—into a truss model ($\rho_{w}\gg \rho_{w,min}$) and a strut-and-tie model ($\rho_{w}\approx \rho_{w,min}$) [55]. In test KH-3, conducted with 1.5 times the minimum shear reinforcement ratio $\rho_{w,min}$ (see Table 1) and exhibiting brittle shear failure (see Figure 9), a compression arch formed, as indicated by a substantial strain increase in the lower longitudinal reinforcement near the supports. Initial signals could be detected at approximately 63% of the failure load, providing an early warning of an impending shear failure. In test KH-5, the warning threshold appeared at approximately 62% of the failure load, whereas in KH-6 it was observed at around 85% of the failure load. The higher threshold in KH-6 is due to its asymmetric shear reinforcement, which increases stiffness, promotes early load redistribution, and thus delays the formation of the compression arch. In contrast, tests KH-2 and KH-4—with significantly higher shear reinforcement ratios (see Table 1)—displayed ductile flexural failure (see Figure 9), as evidenced by large deflections resulting from yielding of the lower longitudinal reinforcement. In test KH-1, conducted without shear reinforcement, evaluation was limited by FOS damage (see Section 3.3.2).

Conversely, measurement data from the FOS in the compression zone do not provide an early warning of an impending shear failure. Nevertheless, fundamental changes can be detected, enabling conclusions about the load-bearing behavior of the beam. It is hypothesized that routing the FOS along the trajectory of the compression arch could yield more accurate results.

## 5. Conclusions and Outlook

Fiber optic sensors offer a promising means to extend service life by monitoring existing concrete bridges with insufficient shear reinforcement or inadequate minimum shear reinforcement ratio, thus ensuring ductile behavior. Since FOSs installed in the tension zone may break prematurely when cracks form, placing FOSs in the compression zone offers a promising alternative for monitoring the load-bearing and deformation behavior of existing concrete structures. Because no studies have fully addressed this setup, shear tests were conducted on reinforced concrete beams with typical shear reinforcement ratios and FOSs installed in both the compression and tension zones at the iBMB, Division of Concrete Construction at the TU Braunschweig. Although the conducted test series comprised only six beams and therefore does not allow for the derivation of universally valid objective diagnostic features or indices, the following findings can be derived from the tests:

1. An FOS, installed on the lower longitudinal reinforcement or in the compression zone, can monitor the fundamental load-bearing behavior of a concrete bridge over long measurement lengths with very high spatial resolution.
2. An FOS installed in the tension zone (on the lower longitudinal reinforcement) enables early crack detection and reliable identification of load redistribution within the structure.
3. An FOS in the compression zone also detects internal load redistribution within the structure and, owing to its extremely low failure probability, enables reliable long-term monitoring of changes in load-bearing behavior. However, it does not provide a reliable early-warning indication of impending shear failure, and clearly attributing strain concentrations to individual cracks remains only partly possible.
4. The robustness of the FOS is particularly significant for long-term monitoring tasks. An FOS in the compression zone offers greater protection against failure compared with an FOS in the tension zone. However, confirming long-term applicability under field conditions will require further investigations.

It is essential to develop a monitoring strategy tailored to the specific problem before any measurements begin. Developing a universally applicable standard sensor arrangement is infeasible. Instead, an analysis of the expected failure mode and the identification of early-warning indicators must be conducted first—if necessary, employing alternative scientific methods [56,57,58]. This article presents characteristic sensor signatures for shear and flexural failure from FOSs installed in the tension and compression zones of beams, providing valuable guidance for interpreting and evaluating measurement data.

Based on these results, additional FOS configurations, e.g., loop-shaped arrangements in highly shear-critical regions and layouts aligned with the compression–strut path, should be examined. Furthermore, additional investigations are required to enable clear and potentially (semi-) automated data evaluation [59,60,61], which is a prerequisite for economically viable practical application. It is also essential for practical applications to investigate the effects of cyclic traffic loading, environmental exposure, and adhesive aging on long-term performance.

Ongoing investigations at the iBMB, Division of Concrete Construction at the TU Braunschweig, are currently being carried out. The primary objective is to evaluate alternative FOS configurations—such as horizontal or inclined, loop-shaped arrangements—in shear-critical regions to further improve the monitoring of shear behavior. The results of these investigations will be reported at a later date.

## Figures and Tables

**Table 1:** Overview of stirrup reinforcements between supports.

Test Specimen	${Ø}_{w}$ [mm]	$s_{w}$ [cm]	$\rho_{w}$ [‰]	$\rho_{w}/\rho_{w,min}$
KH-1	-	-	0	-
KH-2	8	6	8.4	8.1
KH-3	6	20	1.4	1.5
KH-4	8	20	2.5	2.3
KH-5	6	20	1.4	1.8
KH-6	8 (l.)/6 (r.)	6 (l.)/20 (r.)	8.4 (l.)/1.4 (r.)	7.3 (l.)/1.3 (r.)

**Table 2:** Concrete mixture.

Components	Fine Aggregate 0/2 mm	Coarse Aggregate 2/8 mm	Coarse Aggregate 8/16 mm	Cement CEM II/ 52,5R	Water	Admixture Viscocrete
[$kg/m^{3}$]	789	520	561	368	150	3.31

**Table 3:** Mean values of concrete material properties.

Test Specimen	$f_{cm,cyl}$ [N/mm^2^]	$f_{ctm,sp}$ [N/mm^2^]	$E_{cm}$ [N/mm^2^]
KH-1	42.0	3.05	31,100
KH-2	40.7	3.55	31,900
KH-3	45.1	3.35	36,500
KH-4	46.3	3.69	34,700
KH-5	36.3	2.75	27,000
KH-6	56.5	3.93	39,400

**Table 4:** Mean values of reinforcing material properties.

Diameter Ø [mm]	$f_{ym}$ [N/mm^2^]	$f_{tm}$ [N/mm^2^]	$E_{sm}$ [N/mm^2^]
6	561	604	206,200
8	547	585	197,200
20	558	660	200,600

**Table 5:** Failure loads and failure modes of tested beams.

	KH-1	KH-2	KH-3	KH-4	KH-5	KH-6
Failure load $\;F_{u}$ [kN]	145.0	332.9	275.8	339.0	278.4	288.5
Failure mode	shear	flexural	shear	flexural	shear	shear
Shear reinforcement ratio $\rho_{w}/\rho_{w,min}$	-	8.1	1.5	2.3	1.8	7.3/1.3

**Figure A1:**
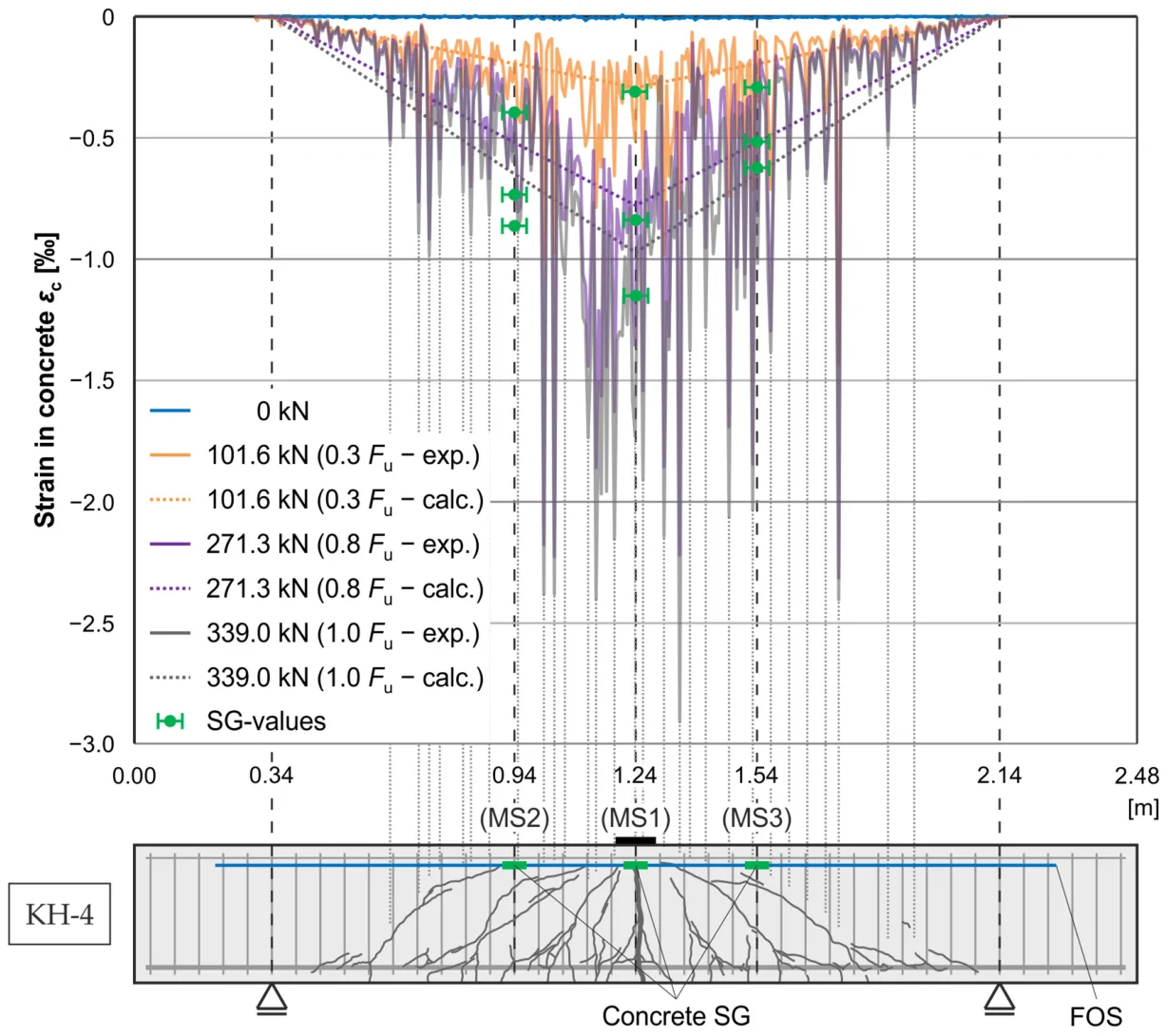
Evaluation of the measured values determined using an FOS and SGs on the concrete surface in the compression zone of beam KH-4 with flexural failure.

**Figure A2:**
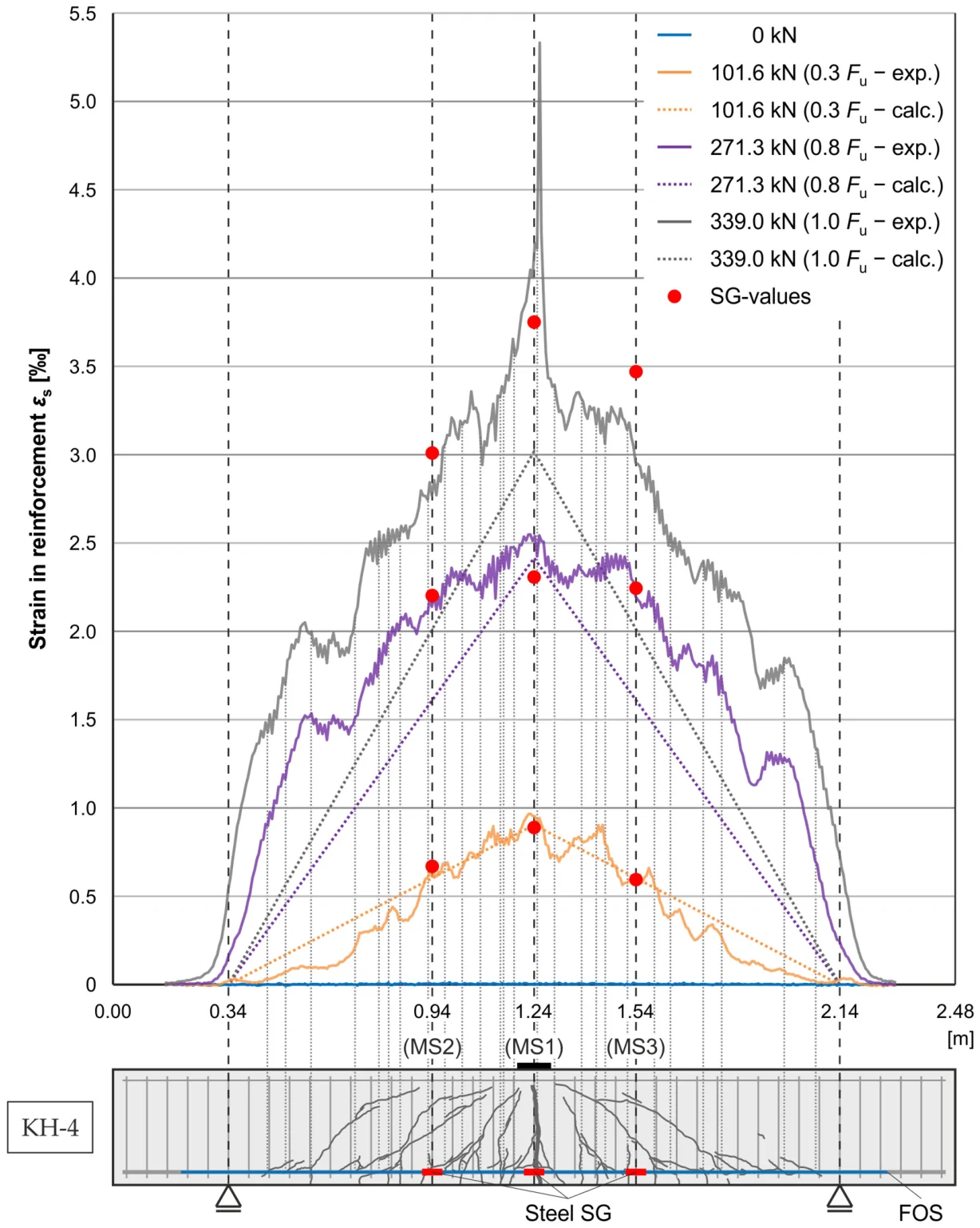
Evaluation of the measured values determined using an FOS and SGs on the lower longitudinal reinforcement in the tension zone of beam KH-4 with flexural failure.

**Figure A3:**
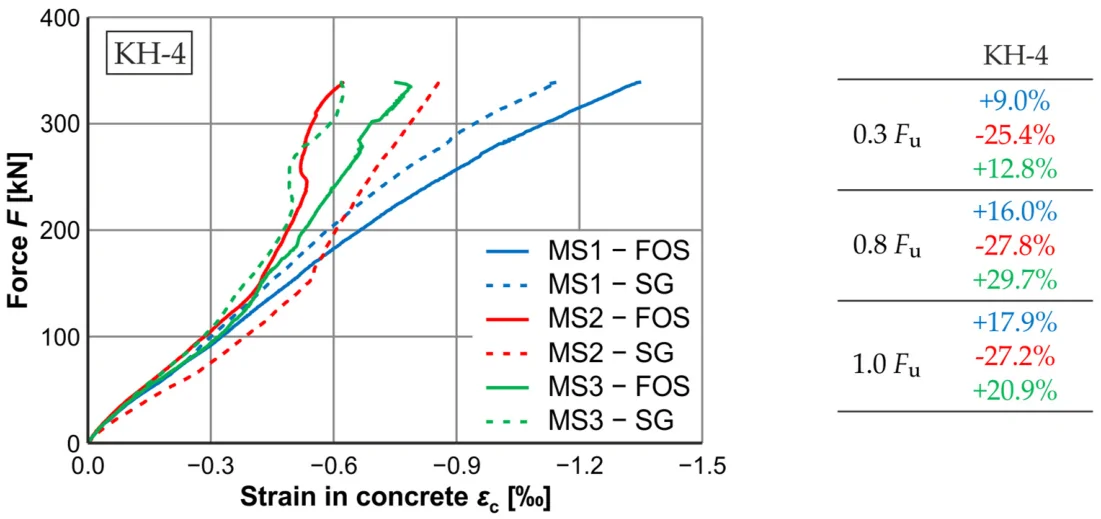
Comparison of strains measured using an FOS and SGs in the compression zone for beam KH-4 and deviations between FOS and SG measurements (MS1: blue; MS2: red; MS3: green).

**Figure A4:**
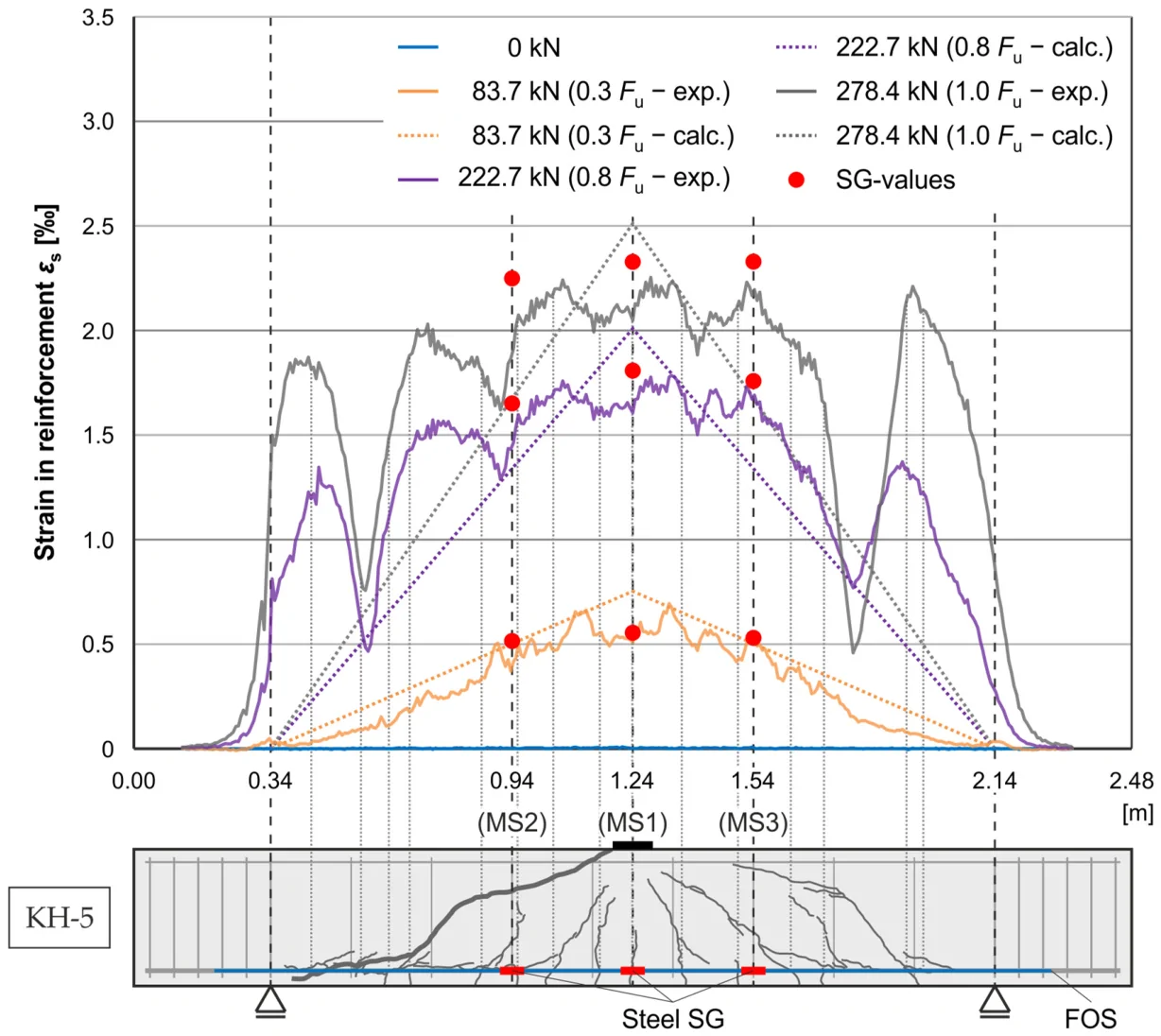
Evaluation of the measured values determined using an FOS and SGs on the lower longitudinal reinforcement in the tension zone of beam KH-5 with shear failure.

**Figure A5:**
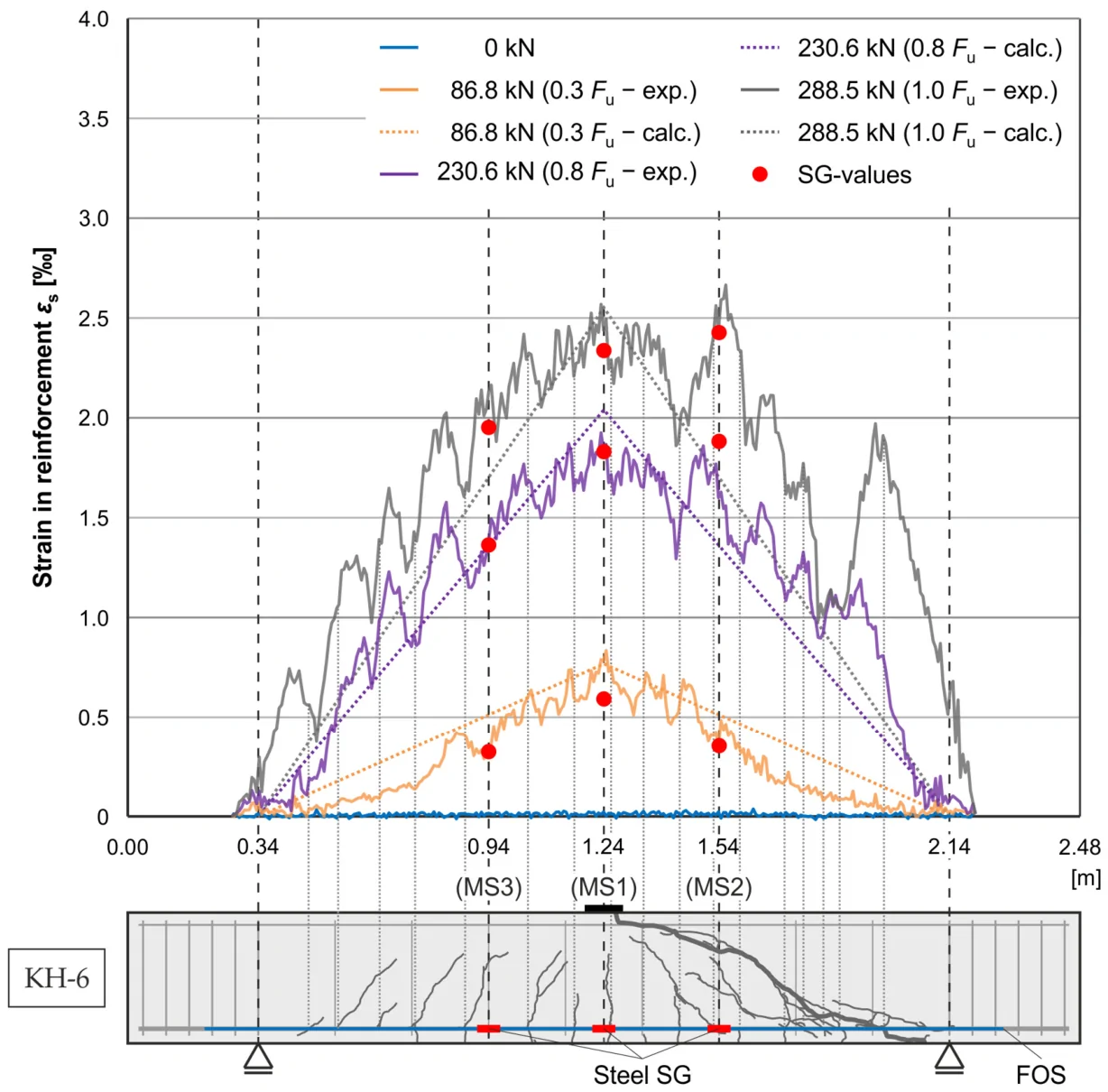
Evaluation of the measured values determined using an FOS and SGs on the lower longitudinal reinforcement in the tension zone of beam KH-6 with shear failure.

## Data Availability

The data that support the findings of this study are available from the corresponding author upon reasonable request.

## Abbreviations

The following abbreviations are used in this manuscript:

$A_{sl}$	Cross-sectional area of the longitudinal reinforcement
b	Width of the cross-section
d	Effective depth of the cross-section
$E_{cm}$	Mean modulus of elasticity of the concrete
$\varepsilon_{c}$	Compressive strain in concrete
$\varepsilon_{c,FOS}$	Compressive strain in concrete at the height of the sensor fiber
$E_{sm}$	Mean modulus of elasticity of reinforcement
$\varepsilon_{s}$	Strain in reinforcement
F	Force applied in the test
$F_{c}$	Concrete compressive force
FOS	Fiber optic sensor
$F_{s}$	Tensile force in the reinforcement
$F_{u}$	Failure load/ultimate force applied in the test
$f_{cm,cyl}$	Mean concrete cylinder compressive strength
$f_{ctm}$	Mean axial tensile strength of concrete
$f_{ctm,sp}$	Mean tensile splitting strength of concrete
$f_{tm}$	Mean tensile strength of reinforcement
$f_{ywk}$	Characteristic tensile strength of stirrup reinforcement
$f_{ym}$	Mean yield strength of reinforcement
h	Height of the cross-section
l	Length of the beam
$l_{eff}$	Effective length of the beam
M	Bending moment
NA	Neutral axis
OFDR	Optical Frequency Domain Reflectometry
$\rho_{w}$	Reinforcement ratio for shear reinforcement
$\rho_{w,min}$	Minimum shear reinforcement ratio according to EC2 [42] and National Annex [43]
SG	Strain gauges
$s_{w}$	Spacing of the shear reinforcement
w	Deflection at midspan
x	Neutral-axis depth measured from the most compressed fiber
z	Inner lever arm of the internal forces
Ø	Diameter of the longitudinal reinforcing bar
${Ø}_{w}$	Diameter of the stirrup reinforcement

## Appendix A

The appendix provides the detailed evaluations of tests KH-4 (FOS in the tension and compression zones, and comparison of strains measured using FOS and SG), KH-5 (FOS in the tension zone), and KH-6 (FOS in the tension zone) to ensure full verifiability.
